# Mutational and Structural Analyses of *Caldanaerobius polysaccharolyticus* Man5B Reveal Novel Active Site Residues for Family 5 Glycoside Hydrolases

**DOI:** 10.1371/journal.pone.0080448

**Published:** 2013-11-20

**Authors:** Takuji Oyama, George E. Schmitz, Dylan Dodd, Yejun Han, Alanna Burnett, Naoko Nagasawa, Roderick I. Mackie, Haruki Nakamura, Kosuke Morikawa, Isaac Cann

**Affiliations:** 1 Institute for Protein Research, Osaka University, Osaka, Japan; 2 Energy Biosciences Institute, University of Illinois, Urbana, Illinois, United States of America; 3 Institute for Genomic Biology, University of Illinois, Urbana, Illinois, United States of America; 4 Department of Microbiology, University of Illinois, Urbana, Illinois, United States of America; 5 Department of Animal Sciences, University of Illinois, Urbana, Illinois, United States of America; Weizmann Institute of Science, Israel

## Abstract

CpMan5B is a glycoside hydrolase (GH) family 5 enzyme exhibiting both β-1,4-mannosidic and β-1,4-glucosidic cleavage activities. To provide insight into the amino acid residues that contribute to catalysis and substrate specificity, we solved the structure of CpMan5B at 1.6 Å resolution. The structure revealed several active site residues (Y12, N92 and R196) in CpMan5B that are not present in the active sites of other structurally resolved GH5 enzymes. Residue R196 in GH5 enzymes is thought to be strictly conserved as a histidine that participates in an electron relay network with the catalytic glutamates, but we show that an arginine fulfills a functionally equivalent role and is found at this position in every enzyme in subfamily GH5_36, which includes CpMan5B. Residue N92 is required for full enzymatic activity and forms a novel bridge over the active site that is absent in other family 5 structures. Our data also reveal a role of Y12 in establishing the substrate preference for CpMan5B. Using these molecular determinants as a probe allowed us to identify Man5D from *Caldicellulosiruptor bescii* as a mannanase with minor endo-glucanase activity.

## Introduction

Biofuels generated from the deconstruction and fermentation of lignocellulosic biomass have the potential to become an important and sustainable alternative energy source [1]. An important step in developing cost-effective biofuels production is to identify enzymes that efficiently degrade plant biomass under conditions suitable for biotechnology processing. In this regard, thermophilic glycoside hydrolases have considerable utility in the emerging biofuels industry due to their capacity to improve the efficiency of polysaccharide hydrolysis and to mitigate costs associated with enzymatic saccharification [2]. The major substrates that are targeted for biofuels production include the plant structural polysaccharides, cellulose and hemicellulose. Hetero-mannans represent a significant component of plant hemicelluloses and consist of a linear backbone of β-1,4-linked mannose sugars (mannans) or a combination of glucose and mannose sugars (glucomannans) that may be substituted with galactosyl groups (i.e., galactomannans or galactoglucomannans) [2]. Enzymes involved in the degradation of mannans include β-mannanases that hydrolyze internal linkages within the mannan backbone, β-mannosidases that cleave mannose from oligosaccharides and acetyl esterases and α-galactosidases that cleave acetyl and galactosyl substituents attached at various positions along the β-1,4-linked mannose chain [3].

*Caldanaerobius polysaccharolyticus* is a thermophilic bacterium that appears capable of efficient mannan fermentation. The draft genome sequence of *C. polysaccharolyticus* revealed two genes, *man5A* and *man5B* that encode glycoside hydrolase (GH) family 5 β-mannanases. CpMan5A is a multidomain β-mannanase [4] containing two family 16 carbohydrate binding modules (CBMs) with specificity for β-1,4-linked cello- and manno-configured oligosaccharides [5]. CpMan5B is an intracellular enzyme that possesses enzymatic activity primarily for manno-oligosaccharides but also cello-oligosaccharides [6]. Recent studies have provided insight into the amino acid residues that determine substrate specificity for GH5 enzymes with β-mannanase and endo-glucanase activities [7]. However, despite exhibiting the two activities, the amino acid residues are different for CpMan5B than for other dual-specifity GH5 enzymes. To provide insight into the molecular underpinnings that mediate substrate specificity for CpMan5B, we solved the crystal structure of CpMan5B to 1.6 Å resolution. The structure guided mutational analyses of active site residues that yield insight into the molecular basis for substrate specificity in this enzyme.

## Materials and Methods

### Compounds and reagents

Manno-oligosaccharides (mannobiose [M2], mannotriose [M3], mannotetraose [M4], mannopentaose [M5], and mannohexaose [M6]) and cello-oligosaccharides (cellotriose [G3], cellotetraose [G4], cellopentaose [G5], and cellohexaose [G6]) were purchased from Megazyme (Bray, Ireland). Cellobiose (G2) was purchased from Sigma-Aldrich (St. Louis, MO). Protein molecular weight markers for sodium dodecyl sulfate-polyacrylamide gel electrophoresis (SDS-PAGE) were obtained from Bio-Rad (Hercules, CA). High-performance liquid chromatography (HPLC) grade sodium acetate trihydrate was purchased from EMD Chemicals (Philadelphia, PA). All other reagents were purchased from Fisher Scientific (Hanover Park, IL) and were of the highest purity available.

### Cloning and site-directed mutagenesis

Amplification of the *man5B* ORF (GenBank Accession ID: HM241690; protein ID: ADK22147) from *C. polysaccharolyticus* genomic DNA and cloning into the pET-46b Ek/LIC vector (Novagen, San Diego, CA) were described previously [6]. Amino acid substitutions (Y12A, Y12F, Y12Q, H84A, H84E, H84M, H84Q, N92A, N136A, R196A, and R196H) were constructed by introducing site-specific mutations into the coding sequence of *man5B* using either the QuikChange II or QuikChange Lightning Multi Site-Directed Mutagenesis Kit (Agilent Technologies, Inc., Santa Clara, CA) according to the supplier’s instructions. The pET46-Man5B plasmid [6] was used as the template, and mutagenic primers (Table S1 in File S3) were designed with the online QuikChange Primer Design program (https://www.genomics.agilent.com). 

The PCR product (12.5 μL) was incubated with methylation-dependent restriction enzyme DpnI at 37 °C for 8 hours to digest the parental plasmid DNA. *Escherichia coli* XL10 competent cells were transformed with the product from the DpnI restriction digestion and plated onto lysogeny broth (LB) solidified with Bacto-agar (Difco) containing 100 μg mL^-1^ ampicillin sodium salt, and the plates were incubated at 37 °C overnight. Individual colonies were cultivated in LB medium supplemented with ampicillin until stationary phase was reached, and plasmids were extracted using a QIAprep^®^ Spin Miniprep Kit (Qiagen, Valencia, CA). The presence of the site-directed mutations was confirmed by DNA sequencing (W.M. Keck Center for Comparative and Functional Genomics, University of Illinois at Urbana-Champaign). 

*Caldicellulosiruptor bescii* DSM6725 ORF0234 (Athe_0234; GenPept Accession ID: YP_002572157) was amplified with PrimeStar^®^ DNA polymerase (Takara Bio Inc., Shiga, Japan) using primers Cb234F and Cb234R (Table S1 in File S3). The PCR product (*cb234*) was extracted from a 1% agarose gel using a QIAquick^®^ Gel Extraction Kit (Qiagen, Valencia, CA). The purified PCR product was treated with T4-DNA polymerase and dATP, annealed to the pET46b Ek/LIC vector (Novagen, San Diego, CA) according to the manufacturer’s instructions, and transformed into *E. coli* strain XL10 (Stratagene, La Jolla, CA). The transformed cells were plated on LB agar plates supplemented with ampicillin. Plasmids were extracted from the transformed cells, and the integrity of *cb234* was confirmed by DNA sequencing as described above. 

DNA oligonucleotide primers were purchased from Integrated DNA Technologies (Coralville, IA, USA). *E. coli* BL21-CodonPlus(DE3)-RIPL cells (Agilent Technologies, Inc., Santa Clara, CA) were used for protein production. *E. coli* strains XL10 and DH5α were used to propagate the recombinant plasmids. 

### Recombinant protein production and purification

*E. coli* BL21-CodonPlus(DE3)-RIPL cells harboring a recombinant plasmid bearing either the gene for wild-type CpMan5B, mutants of CpMan5B, or CbMan5D were cultured in 10-mL LB containing 100 μg mL^-1^ sodium-ampicillin and 50 μg mL^-1^ chloramphenicol for approximately 8 hours at 37 °C with vigorous shaking. The initial 10-mL cultures were used to inoculate LB (1 L) containing the two antibiotics, at the same concentrations stated above, in a 2.8-L Fernbach flask. The cultures were incubated at 37 °C at 200 rpm until the absorbance at 600 nm reached ≈0.3 (after approximately 2.5 hours). Gene expression in *E. coli* cultures was then induced with the addition of IPTG (0.1 mM, final). The temperature was decreased to 16 °C, and the cultures were allowed to incubate for an additional 16 hours at 200 rpm. Cell pellets were harvested from the cultures by centrifugation at 4,651 × *g* for 30 minutes at 4 °C, washed with 35 mL binding buffer (50 mM Tris, 300 mM NaCl, pH 7.5), and frozen as cell pellets at -80 °C.

Frozen cell pellets were thawed and re-suspended in 35-mL ice-cold binding buffer and passed through an EmulsiFlex C-3 homogenizer (Avestin, Ottawa, Canada) to rupture the cells. The cell lysates were clarified by centrifugation at 12,857 × *g* for 20 min at 4 °C, and the supernatant containing the soluble fraction was recovered.

For purification of wild-type or mutant CpMan5B and CbMan5D, the cell-free extracts were incubated at 65 °C for 30 min and centrifuged at 12,857 × *g* for 20 min at 4 °C to pellet the precipitated heat-labile *E. coli* proteins. Cloning of the genes fused each to a hexa-histidine tag encoded in the plasmid. Thus, each protein was purified from the resulting supernatants using Talon metal affinity resin (Clontech, Mountain View, CA) according to the manufacturer’s instructions. Briefly, the resin was incubated with the lysate for one hour to allow binding of the recombinant protein. The resin was then washed with ten column volumes of binding buffer, and the protein was eluted with elution buffer (50 mM Tris, 300 mM NaCl, 250 mM imidazole pH 7.5). The buffer of the eluate containing the recombinant protein was exchanged by concentrating the protein in Spin-X UF 20-mL centrifugal concentrators with molecular mass cutoffs of 10 kDa (Corning, Lowell, MA). The protein in the eluate buffer was twice diluted >20-fold into protein storage buffer (50 mM Tris, 150 mM NaCl, pH 7.5). 

Protein concentrations were determined using a spectrophotometer according to a standard method [8]. Briefly, the absorbance at 280 nm (A_280_) of the recombinant protein solutions was determined using a NanoDrop 1000 from Thermo Fisher Scientific Inc. (Waltham, MA). The molecular masses of the recombinant proteins were predicted using the online ProtParam tool (http://web.expasy.org/protparam/). The values of A_280_, molecular mass, and extinction coefficient (Table S2 in File S3) were used to calculate the concentration of each purified protein.

### Crystallization, data collection and model refinement

Initial screening of crystallization conditions for CpMan5B was performed by the sitting-drop vapor diffusion method using a nanodrop dispenser, Mosquito (TTP Labtech), and two crystallization screening kits, Crystal Screen and INDEX (Hampton Research). Each crystallization drop was made by mixing 200 nL of the concentrated protein solution and an equal volume of reservoir solution. Plate-like crystals were obtained from a drop with a reservoir containing 0.2M ammonium phosphate monobasic, 0.1 M Tris (pH 8.5) and 50% (v/v) (+/-)-2-methyl-2,4-pentanediol (MPD) (Crystal Screen H7). Diffraction quality crystals were reproducibly obtained by the hanging-drop vapor diffusion method in a larger scale manual setup, where 1 μL of the protein solution was mixed with an equal volume of the reservoir solution and equilibrated against 500 μL of the reservoir at 20 °C. Better crystals grew to a maximum dimension of 100 μm in two weeks. 

X-ray Diffraction data were collected at BL38B1 in SPring-8 (Harima, Japan). The CpMan5B crystals were picked up from the crystallization drops and then flash-cooled in a liquid nitrogen gas stream. No cryoprotection was required because the crystallization drops contained a high concentration of MPD. The collected data were processed using the HKL2000 package [9].

The CpMan5B structure was determined by the molecular replacement method with the program CNS [10], using the atomic coordinates of *Thermotoga maritima* endo-glucanase (PDB code 1VJZ), of which sequence identity is 41%, as a probe. The crystals belong to the monoclinic space group *P*2_1_, with the unit cell dimensions of *a* = 50.133 Å, *b* = 147.642 Å, *c* = 55.422 Å, and β = 104.51°, and contained two CpMan5B molecules in the asymmetric unit. The two correctly positioned molecules were refined with CNS and O [11] until convergence. The crystallographic data and refinement statistics are summarized in Table 1. The three dimensional coordinates are deposited in the Protein Data Bank under code 3W0K.

**Table 1:** Crystallographic data collection and refinement.

***Data collection***	
Space group	*P*2_1_
Unit cell constants (Å)	
*a*	50.133
*b*	147.642
*c*	55.422
β	104.51°
Wavelength (Å)	1.0000
Resolution (Å)**^a^**	50.0-1.60 (1.66-1.60)
No. of total reflections	387392 (37733)
No. of unique reflections	102028 (10198)
Completeness (%)	99.8 (100.0)
*I*/σ(I)	16.4 (9.5)
*R*_merge_ (%) **^b^**	3.6 (19.1)
***Refinement***	
Resolution range (Å)	50.0-1.60
*R*_work_ **^c^** / *R*_free_ **^d^**	17.6/18.7
Number of atoms	
Protein	2710
Ligand (Tris)	16
Water	626
Average *B*-factor (Å)	
Protein	15.98
Ligand (Tris)	18.79
Water	26.77
r.m.s.d.	
Bond lengths (Å)	0.006
Angles (deg)	1.3
PDB code	**3W0K**

a Values in parentheses correspond to the last shell. Σ

b *R*_merge_ = (Σ I_*I*_ < I_*I*_ >|)/Σ _*I*_|I_*I*_|, where < I_*I*_ > is the mean I_*I*_ over symmetry-equivalent reflections.

c *R*_work_ = Σ|*F*_obs_–*F*_calc_|/S |*F*_obs_|, where *F*_obs_ and *F*_calc_ are the observed and calculated structural factors, respectively.

d *R*_free_ = Σ|*F*_obs_–*F*_calc_|/Σ|*F*_obs_| for 5% of the data not used at any stage of structural refinement.

### Biochemical assay of wild-type and mutant CpMan5B and CbMan5D

Enzymatic activities of wild-type CpMan5B and its derivative mutant proteins were determined at 65 °C in 50 mM sodium citrate buffer containing 150 mM NaCl with pH 5.5 in accordance with the previously reported temperature and pH optima for the wild-type enzyme [6] using M6 and G6 as substrates. The enzymatic activity of CbMan5D was assayed in the same buffer as wild-type CpMan5B but at 75 °C. For assays to be analyzed by high-performance anion exchange chromatography with pulsed amperometric detection (HPAEC-PAD), the enzymes were added to the mixtures at a final concentration of 0.5 μM and incubated for 8 hours in a total volume of 50 μL with G6 (5 mg mL^-1^) as substrate or at a final concentration of 0.1 μM and incubated for 10 minutes in a total volume of 30 μL with M6 (5 mg mL^-1^) as substrate. The reactions were heat-inactivated for 10 minutes at 99.9 °C, diluted into distilled, deionized water (9-fold for G6 reactions; 16-fold for M6 reactions), and centrifuged at 15,871 × *g* for 5 minutes. The lengths of reaction time were selected from preliminary experiments performed with wild-type CpMan5B enzyme that identified a period in which production of the mono-, di-, and tri-saccharides was linear over time (data not shown).

The end products were analyzed by HPAEC-PAD using a System Gold^®^ HPLC instrument from Beckman Coulter (Fullerton, CA) equipped with a CarboPac PA1 guard column (4 × 50 mm), a CarboPac PA1 analytical column (4 × 250 mm) from Dionex Corporation (Sunnyvale, CA), and a Coulochem^®^ III electrochemical detector from ESA Biosciences (Chelmsford, MA). The elution condition for cello-oligosaccharides was a linear gradient from 0-150 mM sodium acetate in 100 mM NaOH over 15 min. The elution condition for manno-oligosaccharides was a linear gradient from 0-50 mM sodium acetate in 100 mM NaOH over 30 min [12,13]. Eluted saccharide products were identified and quantified by comparing peak retention times and peak areas from the chromatographs of commercially available mono- and oligo-saccharides as standards.

## Results

### Overall structure of CpMan5B

**Figure 1:**
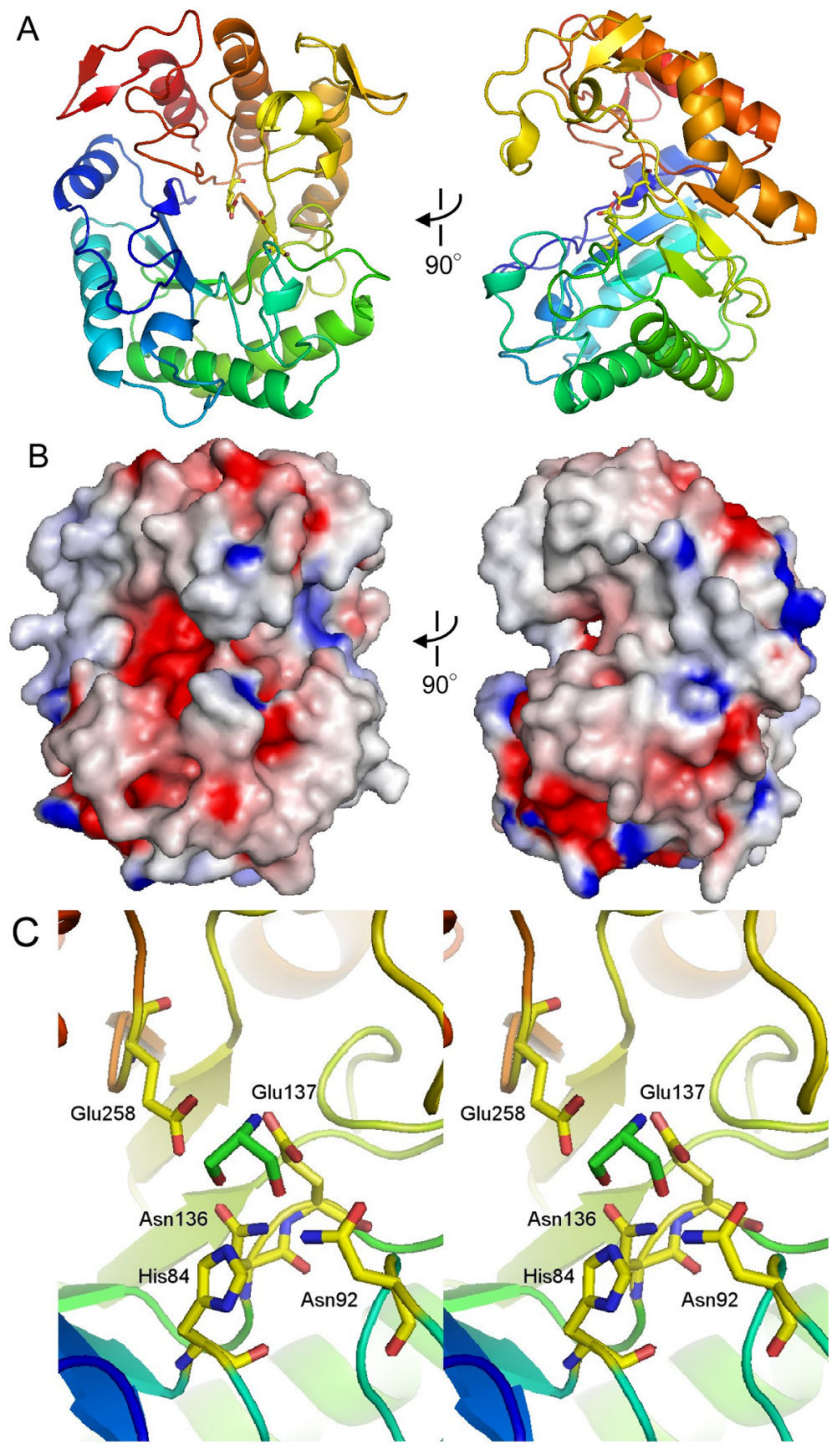
Crystal structure of *C*. ***polysaccharolyticus* Man5B**. A, The structure is shown as a ribbon representation with a color spectrum from blue (N-terminus) to red (C-terminus). B, Orthogonal views of the molecular surfaces of CpMan5B colored according to the electrostatic potential. C, Close-up view of the Tris molecule bound to the active site of CpMan5B. The Tris molecule and the contacting residues are highlighted by a stick representation.

The structure of CpMan5B was determined by the molecular replacement method and refined at 1.6 Å resolution (Figure 1). The protein is well-ordered in the crystal, and, therefore, almost all amino acid residues were visible in the electron density map, except for a short segment encompassing residues 212-216 of chain B. There are two CpMan5B molecules in the asymmetric unit, and the two molecules exhibit almost the same structure, with a relative RMSD of 0.21 Å for the corresponding 325 Cα atoms. CpMan5B folds into an (α/β)_8_ barrel with a deep active site cleft on the carboxy-terminal side of the central β-sheet, which is typical for many carbohydrate hydrolyzing proteins (Figure 1A). Figure 1B shows the molecular surfaces of CpMan5B colored according to the electrostatic potential. The substrate-binding cleft is widely open at both the non-reducing and reducing ends toward solvent, which is a typical feature for endo-acting carbohydrate degrading enzymes.

Incidentally, a Tris molecule, which was supplied from the crystallization reservoir solution, is tightly bound to the active site, and forms hydrogen bonds with five amino acid residues, H84, N92, N136, E137 and E258 (Figure 1C). Residues E137 and E258 were previously determined to be the catalytic residues by sequence alignment and enzymatic assays of site-directed mutants [6], and roles in catalysis for residues equivalent to H84 and N136 have been described for other enzymes [14-17]. In particular, N92 is positioned on the β4-α4 loop, which is usually disordered in the related enzyme structures because of potential flexibility. In the CpMan5B structure, however, this loop is well ordered, possibly due to the hydrogen bond between N92 and the bound Tris molecule.

The structure of CpMan5B is similar to GH5 mannanases and endo-glucanases with known structures. A search of the Dali Database [18] revealed that CpMan5B exhibits highest structural similarity to the mannan-inducible [19] *Thermotoga maritima* glucomannanase [20] (Tm1752) (1VJZ; 41% amino acid identity, RMSD 1.7 Å for corresponding 325 Cα atoms), although, to our knowledge, mannanase activity of Tm1752 has not been demonstrated *in vitro*. CpMan5B exhibits lower similarity to a *Clostridium thermocellum* cellulase (CtCel5C) (1CEN; 28%, 2.2 Å for 334 Cα atoms) [21], the dual specificity mannanase/endo-glucanase from *T. maritima* (Tm1751; TmCel5A) (3AMD; 23%, 2.3 Å for 312 Cα atoms) [22], and an endo-glucanase from the thermophilic bacterium, *Fervidobacterium nodosum* (3RJY; 22%, 2.4 Å for 308 Cα atoms) [23]. 

### Amino acid determinants for binding gluco-configured substrates

**Figure 2:**
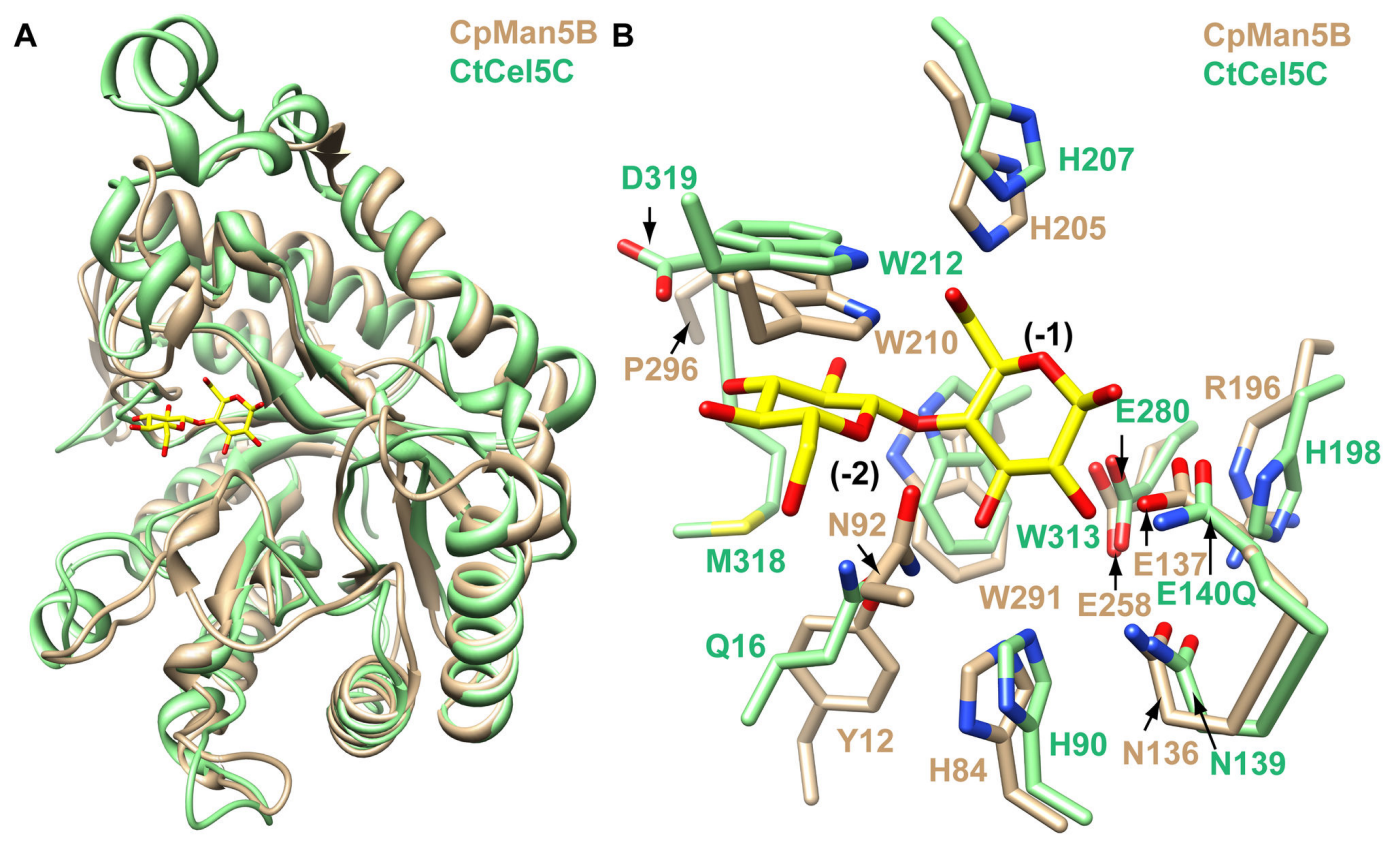
Structural comparison of CpMan5B with *Clostridium* *thermocellum* cellulase (CtCel5C). A, The CtCel5C-cellobiose complex (green) is superimposed onto CpMan5B (tan). B, Close-up view of the active site. The figure is drawn in the same orientation as in A. The cellobiose molecule bound to CtCel5C is shown in a stick model colored yellow and red for carbon and oxygen atoms, respectively. Contacting residues of CtCel5C (green), and the corresponding residues of CpMan5B (tan) are shown in panel B. Sugar subsites are indicated in black font.

Superposition of the *Clostridium thermocellum* Cel5C-cellobiose structure (PDB 1CEN) [21] with CpMan5B (Figure 2) revealed overall conservation in protein fold (Figure 2A). In the CtCel5C-cellobiose complex, two β-1,4-linked glucose molecules occupy the -2 and -1 subsites (according to the nomenclature by Davies et al. [24]) in the active site, and most of the residues contacting the bound sugars are conserved in CpMan5B, possibly providing an explanation for the endo-glucanase activity observed for CpMan5B.

At subsite -1 of CtCel5C, the enzyme makes a number of hydrogen bond contacts to the glucose moiety. These contacts include E140 and E280 with the C1 and C2, hydroxyls, respectively, and bidentate hydrogen bonds to the C3 hydroxyl mediated by Q16 and H90 and to the C6 hydroxyl mediated by W212 and H207. These residues are present in CpMan5B as E137, E258, Y12, H84, W210, and H205, respectively (Figure 2B). With the exception of Q16/Y12, these residues are conserved in GH5 enzymes and are unlikely to significantly alter substrate specificity. At subsite -2, the W313 sidechain Nε and M318 mainchain O atoms form hydrogen bonds with the C2 hydroxyl. It is possible that CtCel5C W313 could also hydrogen bond with an axial OH group at C2 of a mannose moiety, if mannose were bound to the subsite -2 instead of glucose at a similar location and with a similar conformation. Three other hydrogen bond contacts in the -2 subsite include D319 with the C3 hydroxyl and Q16 with the ring oxygen and the C6 hydroxyl of the glucose moiety. Amino acid residue W313 is conserved in CpMan5B as W291, but D319 and Q16 are replaced with P296 and Y12 in CpMan5B, respectively. In addition, two tryptophan residues (W313 and W212) in CtCel5C are co-planar to the sugar residues in the -1 and -2 subsites and thereby help to stabilize these sugars by hydrophobic stacking interactions. These residues are conserved in CpMan5B as W291 and W210. Thus, most of the key residues that stabilize the cellobiose molecule in the CtCel5C-cellobiose structure are conserved in CpMan5B. 

Despite the similarities, several key differences at the -1 and -2 binding subsites could help to explain the differences in specificity between CtCel5C and CpMan5B. The β8-α8 loop, on which CtCel5C M318 and D319 are located, is shorter in CpMan5B. Therefore, the hydrogen bonds between the β8-α8 loop and the sugar occupying subsite -2 in CtCel5C would be absent in CpMan5B, unless a large conformational change of the loop occurs upon substrate binding. Although Q16 of CtCel5C is replaced by Y12 in CpMan5B, it is located in a similar spatial position so that the hydroxyl group of the tyrosine residue could hydrogen bond with the C3 hydroxyl of the -1 subsite-bound sugar, but not with the ring oxygen of the -2 subsite-bound sugar.

Another difference between the two active sites is the presence of N92 in CpMan5B which is contained on the loop between β-strand 3 and α-helix 3. The side chain of N92 faces towards the active site and is positioned to make hydrogen bond contacts with the C6 hydroxyl of the glucose moiety at the -2 subsite. Amino acid sequence alignments reveal Q98 as a corresponding residue in CtCel5C, but in the CtCel5C-cellobiose structure, this residue showed poor electron density definition [21], thereby preventing a direct comparison. The large and diverse family of GH5 enzymes was recently categorized into 51 distinct subfamilies [25], and CtCel5C belongs to subfamily GH5_37 while CpMan5B belongs to subfamily GH5_36. The CtCel5C Q98 residue is not conserved in the GH5_37 subfamily (only 1 of 11 unique sequences with identity <99%), but interestingly, the 27 enzymes in the subfamily GH5_36 show strong conservation (89%) of an asparagine at position 92 (CpMan5B numbering, data not shown), suggesting that this residue may fulfill a unique role in this subfamily. Another difference between the active sites of CtCel5C and CpMan5B is the replacement of H198 with R196. In CtCel5C, H198 forms a 2.7 Å hydrogen bond with the catalytic acid-base catalyst, E140, which is mutated to a glutamine in the CtCel5C structure. In the CpMan5B structure, R196 adopts a conformation that permits a hydrogen bond to form between its Nε atom and the Oε_2_ of the catalytic glutamate, E137. Interestingly, this hydrogen bond is also 2.7 Å in length, suggesting that R196 is sufficient to carry out the same function as H198. As discussed later, this observation is of considerable significance as a histidine at this position has been implicated as being critical for catalysis for GH5 enzymes [23].

### Amino acid determinants for binding manno-configured substrates

**Figure 3:**
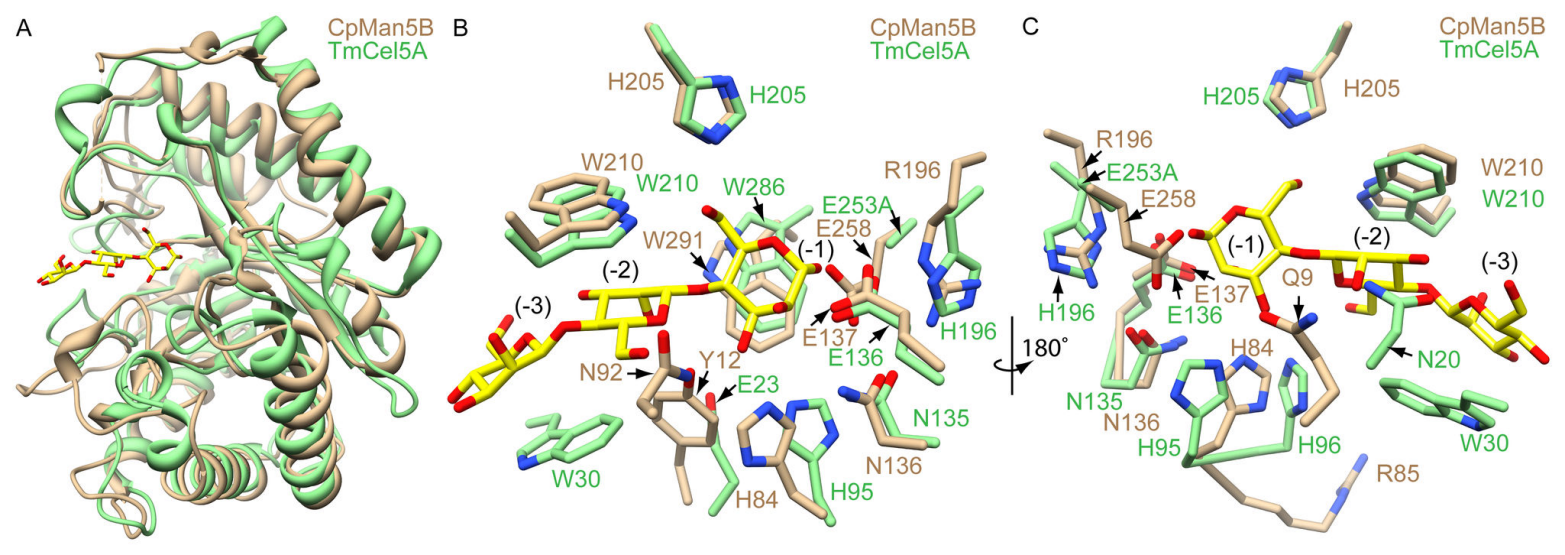
Structural comparison of CpMan5B with *Thermotoga* *maritima* cellulase (TmCel5A). A, The TmCel5A-mannotriose complex (green, PDB ID: 3AZS) is superimposed onto CpMan5B (tan). B, Close-up view of the active site. The mannotriose molecule bound to TmCel5A is shown by a stick model colored yellow and red for carbon and oxygen atoms, respectively. Contacting residues of TmCel5A (green), and the corresponding residues of CpMan5B (tan) are shown in panel B. Sugar subsites are indicated in black font. C. The active site has been rotated 180° around the y-axis relative to the view in panel B.

The superposition of the endo-glucanase CtCel5C and the β-mannanase CpMan5B, which has a minor endo-glucanase activity, suggested that there are several differences in active site residues between the two enzymes. To evaluate whether these residues may correspond to an increased β-mannanase activity, CpMan5B was aligned with the TmCel5A-mannotriose structure [22]. In the TmCel5A-mannotriose complex, three mannose sugars occupy the -3, -2, and -1 subsites in the active site, hereto referred to as Man^(-1)^, Man^(-2)^ and Man^(-3)^ (Figure 3).

In the -1 and -2 subsites, many of the hydrogen bond contacts are conserved in comparison with the TmCel5A-mannotriose structure with several notable differences. These include the replacement of H96 in TmCel5A with R85 in CpMan5B, which appears to point away from the active site, thereby eliminating a hydrogen bond to the Man^(-1)^ C3 hydroxyl. Interestingly, this hydrogen bond contact is replaced by one with Y12 in CpMan5B. Y12 corresponds to E23 in TmCel5A, but the latter did not appear to contact the mannose ligands. Furthermore, the Y12 residue binds to the Man^(-2)^ C2 hydroxyl and hemiacetal oxygen. Notably, N20 in TmCel5A, which forms a hydrogen bond contact with the Man^(-2)^ C2 hydroxyl and has been suggested to be an important determinant of mannanase activity [7], is replaced with Q9 in CpMan5B. The β-carbon of Q9 in CpMan5B is closely approximated (2.0Å) to the α-carbon of N20 in TmCel5A (Figure 3C). It is likely, therefore, that when mannose is bound in the -2 subsite, Q9 forms a hydrogen bond with the C2 hydroxyl. Therefore, in the CpMan5B structure, there is one additional hydrogen bond contact to the Man^(-2)^ sugar mediated by Y12. This residue could in part explain the increased selectivity of CpMan5B for mannans over glucans as compared to TmCel5A.

### Mutational analyses of active site residues

The crystal structure of CpMan5B and comparisons to the three dimensional structures of CtCel5C (subfamily GH5_37) and TmCel5A (subfamily GH5_25) revealed important differences in active site residues. Several residues (Y12, H84, N92, N136, and R196) were changed by site-specific mutagenesis, resulting in thirteen mutant proteins (Figures S1-S2 in File S1, Table S3 in File S3) with relative molecular masses of ≈41kDa, so that their roles in enzyme function could be evaluated.

**Figure 4:**
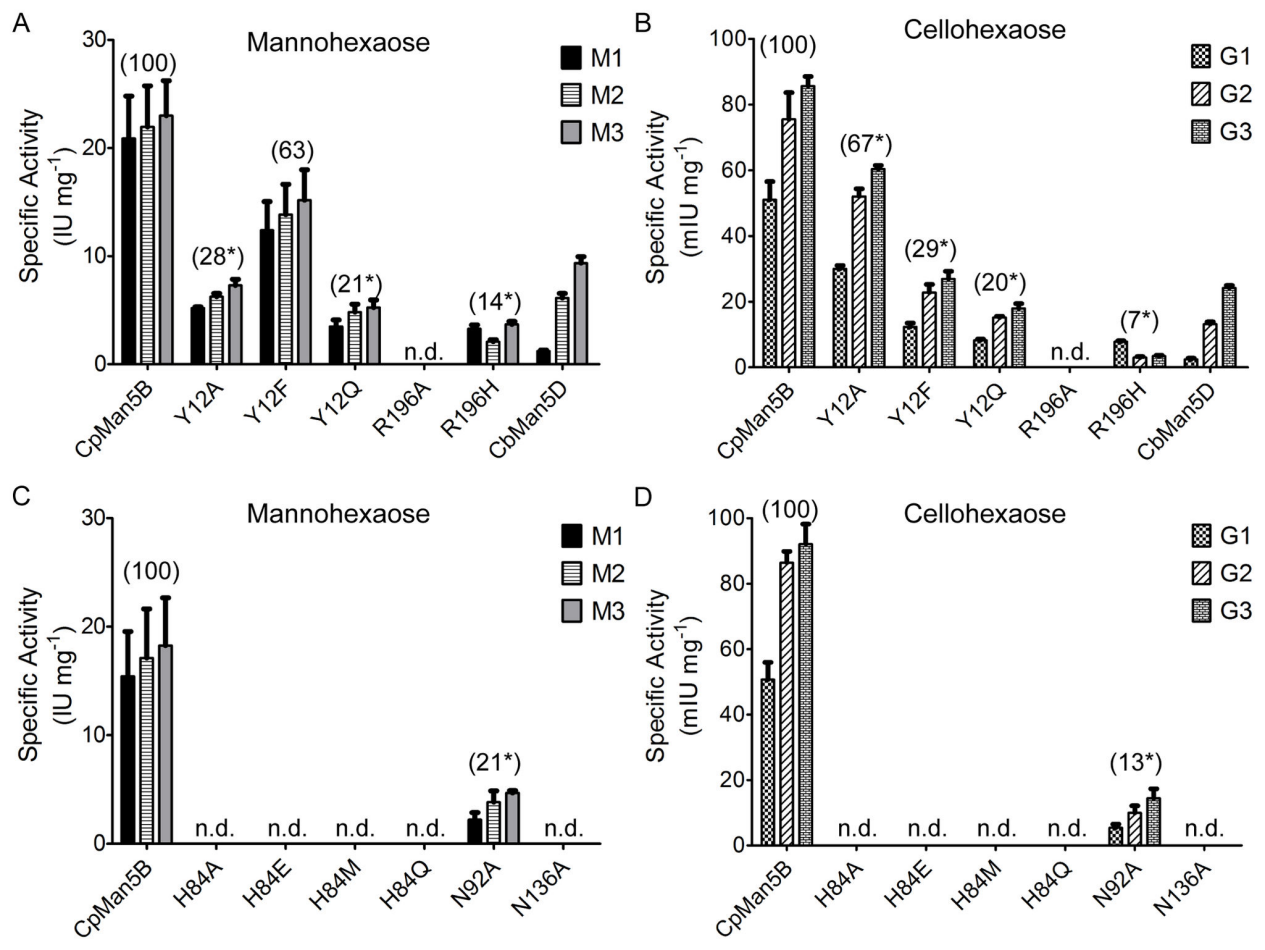
Specific activities of wild-type and mutant CpMan5B enzymes and the CbMan5D enzyme. Rates of product formation from either mannohexaose (Panels A, C) or cellohexaose (Panels B, D) were determined after 10 minutes and 8 hours, respectively, when the rates of product formation by the wild-type enzymes were linear with time. The amino acid changes in the x-axes labels indicate the site-specific mutants of the CpMan5B enzyme. Parenthetical values above the bars show the percentage of wild-type CpMan5B activity, and asterisks indicate that the raw data are significantly different (P<0.05, Student's paired t test) from those of the wild-type CpMan5B enzyme in the same experiment. Abbreviations: M1, mannose; M2, mannobiose; M3, mannotriose; G1, glucose; G2, cellobiose; G3, cellotriose; n.d., end product(s) was not detected under these assay conditions; IU, international units (μmol min^-1^ mg^-1^); mIU, milli-international units (nmol min^-1^ mg^-1^). Lower limits for detection were <2 IU mg^-1^ for mannosaccharides and <2 mIU mg^-1^ for cellosaccharides.

Amino acid substitutions in residues H84, N92, and N136, which form hydrogen bonds with the Tris molecule in the CpMan5B crystal structure, had dramatic effects on enzyme activity for both substrates. Four amino acid substitutions (A, E, M, and Q) at residue H84 and an alanine substitution at residue N136 in CpMan5B virtually eliminated all activity with M6 and G6 (Figure 4) indicating that these two residues are essential to both the mannanase and cellulase activities. As mentioned above, H84 and N136 have been implicated previously in the activities of other β-1,4-endoglucanases. The activities of mutant enzyme CpMan5B^N92A^ were also reduced, although some activity with both substrates was detected.

The residue at position 196 is highly conserved in GH5 enzymes as a histidine [26] and is thought to participate in an electron relay network that is critical for catalysis [7]. Surprisingly, the corresponding residue in CpMan5B is an arginine, and this residue is, in fact, 100% conserved in subfamily GH5_36 (data not shown). Therefore, to evaluate the role of this residue in catalysis, R196A and R196H mutations were made. The R196A mutation eliminated all detectable activity with both substrates, indicating that this residue is essential for catalysis. The CpMan5B^R196H^ enzyme, however, retained some activity with both G6 and M6 substrates (7% and 14% of CpMan5B^WT^, respectively) suggesting that the histidine and arginine are interchangeable at this position.

The structure of CpMan5B and its comparison with the structures of other GH5 enzymes suggested that Y12 could be important for imparting increased β-mannanase activity to CpMan5B. An amino acid sequence alignment of the 27 subfamily GH5_36 enzymes, including CpMan5B, from the GenBank database revealed that this position was replaced with a phenylalanine in 20 enzymes (data not shown). We, therefore, decided to test the effect of a phenylalanine at this position. In addition, less conservative changes, Y12A and Y12Q, were also tested. The Y12A, Y12F, and Y12Q mutations each resulted in moderate losses of activity on both G6 and M6 substrates (Figure 4, Table S4 in File S3). However, CpMan5B^Y12A^ retained 28% of the WT mannanase activity and 67% cellulase activity, but the trend was reversed for the CpMan5B^Y12F^ mutant enzyme, which retained 63% mannanase and 29% cellulase activities. The CpMan5B^Y12Q^ mutant enzyme lost activities proportionately (retaining only ≈20%) for both G6 and M6 substrates. Taken together, these data indicate that the residue at position 12 can alter the selectivity for glucose- or mannose-configured substrates with CpMan5B^Y12F^ retaining the highest preference for mannose-configured substrates. Incidentally, double mutants of the Y12F and Y12Q mutations with the R196H mutation (CpMan5B^Y12F/R196H^ and CpMan5B^Y12Q/R196H^) were constructed, but the activities of these double mutants were very low or undetectable. Therefore, their combined effects on substrate specificity could not be evaluated.

### Identification of C. bescii Man5D as a GH5 enzyme with mannanase and cellulase activities

**Figure 5:**
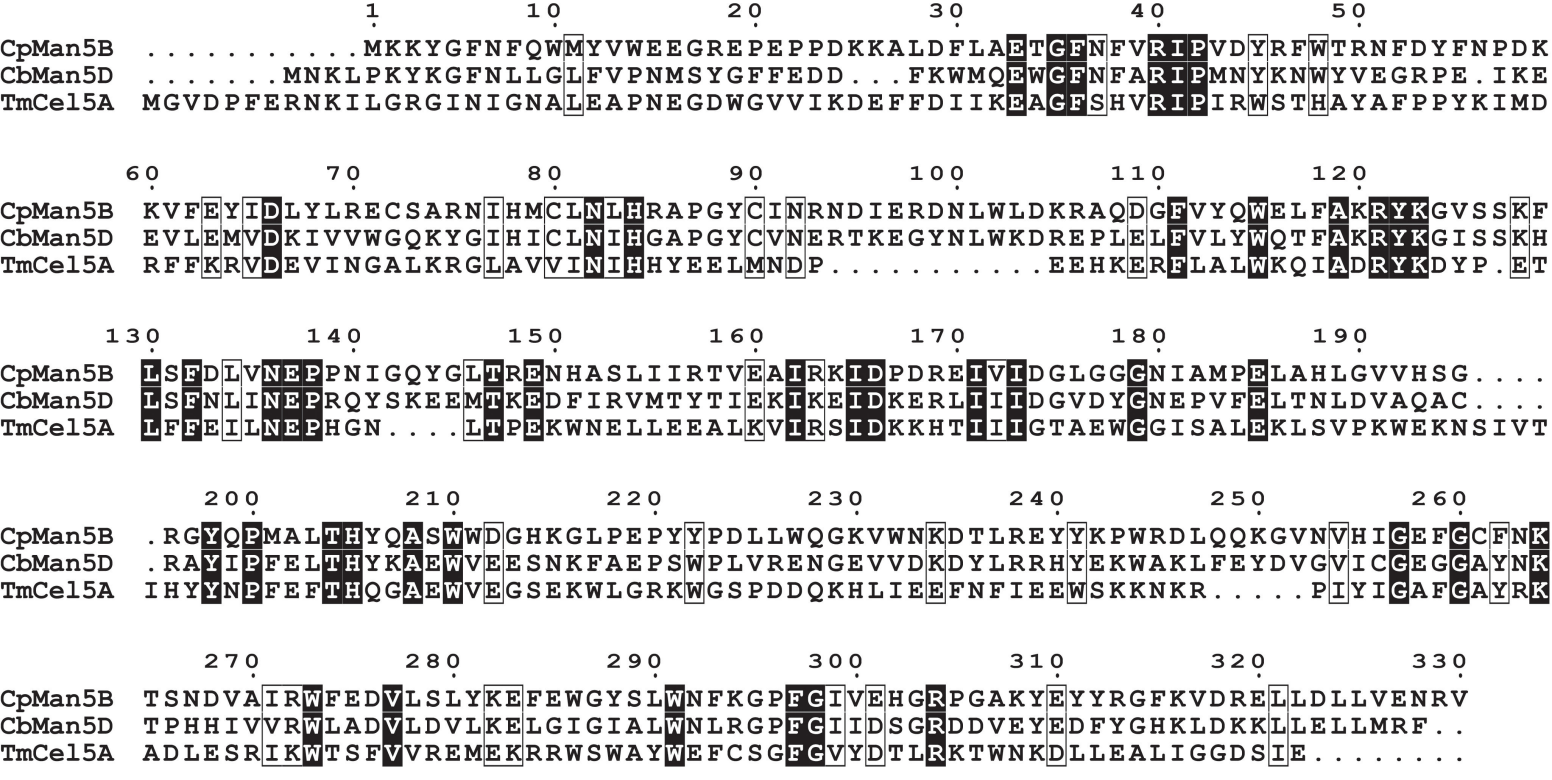
Amino acid sequence alignment of CpMan5B, CbMan5D, and TmCel5A. The three polypeptides were aligned using ClustalW with the Blosum62 similarity matrix (http://www.genome.jp/tools/clustalw/). Boxes indicate similar residues and dark shading indicates identical residues. The GenBank accession numbers for CpMan5B, CbMan5D, and TmCel5A are ADK22147, ACM59384, and AAD36816, respectively.

An alignment of subfamily GH5_36 enzymes revealed that Y12 is conserved in only 5 of 27 enzymes and is replaced frequently by a phenylalanine. Biochemical analysis showed that the Y12F mutant had improved selectivity for manno-oligosaccharides, and we therefore hypothesized that F12 could be used as a probe for enzymes with relatively high mannanase activity. A BLASTp search of the GenBank database revealed ORF0234 (CbMan5D: Genbank Accession ID: ACM59384) from *C. bescii* as a hyperthermophilic putative cellulase with 40.1% identity to CpMan5B. CbMan5D, also in subfamily GH5_36, contains a phenylalanine at the site corresponding to residue Y12 of CpMan5B, but contained identical residues at positions corresponding to H84, N92, N136, and R196. Preliminary analyses of recombinant CbMan5D showed that this enzyme had optimal pH and temperature of 5-5.5 and 75-80°C, respectively (data not shown). CbMan5D was also assayed qualitatively using thin-layer chromatography (TLC, Figure S3 in File S2). CbMan5D hydrolyzed mannose-configured substrates with a degree of polymerization (DP) ≥ 2, which was similar to CpMan5B. CbMan5D also degraded glucose-configured substrates with a DP ≥ 4, differing only slightly from CpMan5B, which could also degrade G3 [6]. Thus, CbMan5D appeared to be a mannanase with some endoglucanase activity similar to CpMan5B. Interestingly, a sequence alignment shows that CbMan5D has an arginine (position 139 according to numbering in Figure 5) which is semi-conserved in the GH5 family and has been shown to bind the +2 mannosyl specifically and to be functionally important [27-29]. The corresponding arginine is not present in CpMan5B (Figure 5).

To compare the relative activities of CbMan5D to those of CpMan5B, CbMan5D was assayed quantitatively using a HPAEC-PAD method similar to that used for CpMan5B, but carried out at 75 °C (Figure 4A-B). The data showed that CbMan5D is also primarily a β-mannanase with a capacity to cleave β-1,4-glucose linkages. To assess substrate specificity, the ratios of β-mannanase:endo-glucanase specific activities for CpMan5B^WT^, CpMan5B^Y12F^ and CbMan5D were calculated as 310, 669, and 420, respectively. The higher ratio values of CpMan5B^Y12F^ and CbMan5D relative to CpMan5B^WT^ suggest that the residue at this position may, in part, influence the relative mannanase:endoglucanase activities in subfamily GH5_36 enzymes.

## Discussion

The GH5 family includes a large number of enzymes with a variety of enzymatic activities [25]. Our previous study identified CpMan5B, an enzyme with β-mannanase and endo-glucanase activities [6] from *C. polysaccharolyticus*, a thermophilic gram positive bacterium that ferments a variety of polysaccharides [30]. Recently, a study suggested that the coexistence of β-mannanase and endo-glucanase activities in GH5 enzymes may be more widespread than originally thought and has perhaps been overlooked due to a failure to routinely test for β-mannanase activity [7]. Using *in silico* and *in vitro* techniques, the investigators identified a set of six residues (N20, E23, P53, H95, H96, and E287 in TmCel5A) that might serve as markers to help predict the presence of dual β-mannanase/endo-glucanase activity in enzymes belonging to the subfamily A4 [7], in a classification system defined by the authors. Subfamily A4 spans several GH5 subfamilies defined by Aspeborg et al. [25] and includes the GH5_25 and GH5_37 subfamilies that are closely related to GH5_36, to which CpMan5B and CbMan5D belong. In CpMan5B, the corresponding six residues are Q9, Y12, P42, H84, R85, and N292, and in CbMan5D, the corresponding residues are L13, F16, P43, H84, G85, and N292 (Figure 5 and Table S5 in File S3). Thus, the enzymes CpMan5B and CbMan5D share only 2 out of 6 of these determinants of catalytic activity. In TmCel5A, an H95A mutation reduced endo-glucanase activity while mannanase activity remained unchanged [7]. In CpMan5B, mutation of the corresponding residue (H84) to four different residues reduced both the mannanase and endo-glucanase activities to <2% of the wild-type enzyme activities. We also identified a role for the poorly conserved residue Y12 of CpMan5B in substrate selectivity. These data may highlight different roles in catalytic activity for the conserved residue H84 and distinct active site residues between the Subfamily A4 and the GH5_36 subfamily. 

**Figure 6:**
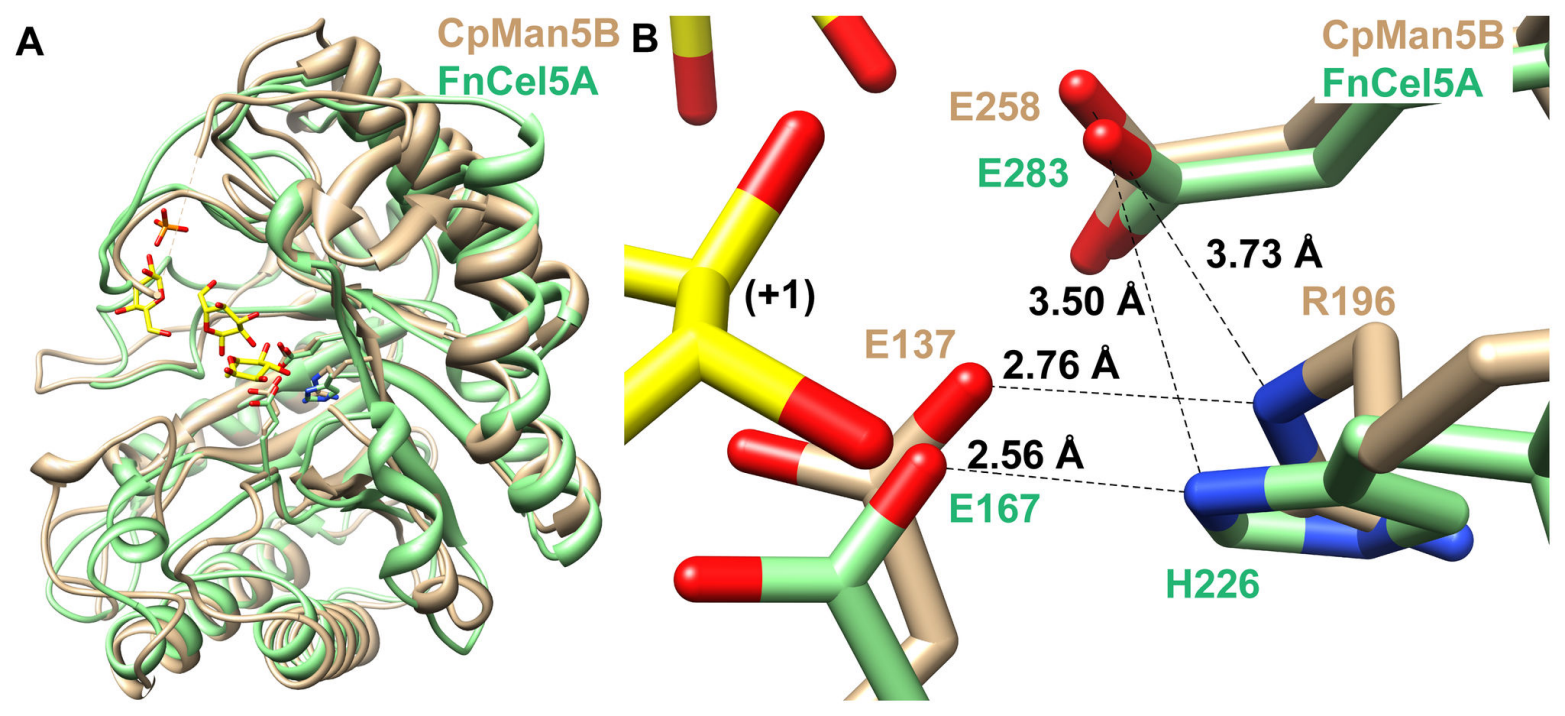
Structural comparison of CpMan5B with *Fervidobacterium* *nodosum* Rt17-B1 endoglucanase (FnCel5A). A, The FnCel5A enzyme (PDB ID: 3RJY) bound to three glucose molecules (yellow and red heteroatoms) and phosphate (orange and red heteroatoms) is superimposed onto CpMan5B (tan). B, Close-up view of the proposed catalytic triad. The figure is drawn in the same orientation as in A. Sugar subsite is indicated in black font.

In GH5 enzymes, it has been proposed that the residue at position 196 (CpMan5B numbering) is strictly conserved as a histidine [31], and mutations at this site completely abolish activity [7,23,31] suggesting that this site is critical for catalysis. Recently, it was suggested that the corresponding residue, H226, in an endo-glucanase from *Fervidobacterium nodosum* Rt17-B1 (FnCel5A) may function in a triad with the two catalytic glutamate residues as an intermediate in electron transfer [23]. Interestingly, every enzyme in subfamily GH5_36 possesses an arginine residue at this position, and a structural alignment of CpMan5B with FnCel5A (PDB code 3RJY; Figure 6) reveals a deviation of just 1.4 Å between the Cα of R196 and H226. Furthermore, the distances from the Nε of R196 in CpMan5B to the catalytic glutamates are similar to the distances from Nδ2 of H226 to the catalytic glutamates in FnCel5A (Figure 6). This observation suggests that R196 can perform a similar function to the corresponding histidine residue in FnCel5A. Indeed, mutation of R196 to alanine completely abolished activity, but mutation of R196 to histidine retained some activity, providing further support that histidine and arginine are interchangeable at this position.

We also observed the presence of N92 in the active site, and an alanine substitution at this site strongly reduced activity of CpMan5B. Interestingly, this residue is strongly conserved in subfamilies GH5_36 and GH5_38 (data not shown) but is not conserved in related subfamilies GH5_4, GH5_25, GH5_37, GH5_39, and GH5_52 [25], indicating that N92 may be a unique characteristic of the GH5_36 and GH5_38 subfamilies. Residue N92 makes a contact with W210, forming a bridge over the active site of CpMan5B, but the significance of this asparagine bridge is currently unknown.

In the current study, we have solved the structure of a β-mannanase/endo-glucanase and established the roles of critical amino acid residues that differ from other characterized GH5 enzymes. In the context of other recent work with dual specificity enzymes, our results highlight the relative plasticity among GH5 enzymes both in terms of substrate specificity and catalysis. From a biotechnological perspective, GH5 enzymes represent an excellent scaffold upon which novel enzyme activities and specificities may be engineered. 

## Supporting Information

File S1 **Figures S1 and S2.** Figure S1. Purification of CpMan5B mutant proteins and CbMan5D. CpMan5B, mutant proteins of CpMan5B, and CbMan5D were expressed recombinantly in *E. coli* and purified from crude cell-free extracts by cobalt metal affinity chromatography. Two μg protein were loaded per well, separated by SDS-PAGE, and stained with Coomassie brilliant blue G-250. Panel A: lane 1, Bio-Rad Broad Range molecular mass standards; lanes 2-9, CpMan5B^WT^, CpMan5B^Y12A^, CpMan5B^Y12F^, CpMan5B^Y12Q^, CpMan5B^R196A^, CpMan5B^R196H^, CpMan5B^Y12F/R196H^, and CpMan5B^Y12Q/R196H^, respectively; lane 10, CbMan5D. Panel B: lane 1 Bio-Rad Broad Range molecular mass standards; lanes 2-8, CpMan5B^WT^, CpMan5B^H84A^, CpMan5B^H84E^, CpMan5B^H84M^, CpMan5B^H84Q^, CpMan5B^N92A^, and CpMan5B^N136A^, respectively. Figure S2. Immunoblot analyses of CpMan5B mutant proteins and CbMan5D. Purified CpMan5B, mutant proteins of CpMan5B, and CbMan5D were loaded (2 μg protein per lane), separated by SDS-PAGE, transferred to a PVDF membrane and analyzed by Western blot. Panel A: lane 1, Bio-Rad Precision Plus Protein™ Kaleidoscope Standards; lanes 2-9, CpMan5B^WT^, CpMan5B^Y12A^, CpMan5B^Y12F^, CpMan5B^Y12Q^, CpMan5B^R196A^, CpMan5B^R196H^, CpMan5B^Y12F/R196H^, and CpMan5B^Y12Q/R196H^, respectively; lane 10, CbMan5D. Panel B: lane 1, Bio-Rad Precision Plus Protein™ Kaleidoscope Standards; lanes 2-8, CpMan5B^WT^, CpMan5B^H84A^, CpMan5B^H84E^, CpMan5B^H84M^, CpMan5B^H84Q^, CpMan5B^N92A^, and CpMan5B^N136A^, respectively.
(DOCX)

File S2 **Figure S3.** Figure S3. Activity of CbMan5D with cello- and manno-oligosaccharides as detected by thin layer chromatography. CbMan5D (2.5μM) was reacted with 5 mg mL^-1^ substrate for 12 hours at 75 °C. One microliter of the reaction products was spotted in each lane. Minus (-) and plus (+) signs indicate the absence or presence of CbMan5D, respectively. Panel A shows the reactions containing manno-configured saccharides (M1-M6). Panel B shows reaction products of gluco-configured saccharides (G1-G6). Standards containing 1 μg each oligosaccharide (M1-M6, Panel A; G1-G6, Panel B) were loaded in lanes at both ends of the plate.
(DOCX)

File S3 **Supplementary Tables S1, S2, S3, S4, S5.** Table S1. Primer sequence used for gene cloning and mutagenesis. Table S2. Protein properties used for determining protein concentrations. Table S3. Analysis of CD spectra for CpMan5B WT and mutant proteins using DICHROWEB. Table S4. Effects of different mutations on specific activities compared with that of the wild-type CpMan5B protein. Table S5. Comparison of residues in TmCel5A that affect dual mannanase/endoglucanase activity to residues of GH5_36 enzymes.
(DOCX)

File S4 **RCSB PDB Validation Report.**
(PDF)

File S5 **RCSB PDB Validation Report Summary.**
(PDF)
