# Supplementary material for: Mutational and Structural Analyses of Caldanaerobius polysaccharolyticus Man5B Reveal Novel Active Site Residues for Family 5 Glycoside Hydrolases
Source: PLoS One. 2013 Nov 20;8(11):e80448. doi: 10.1371/journal.pone.0080448 (PMC3835425; doi:10.1371/journal.pone.0080448)
Supplement: File S1 — Figures S1 and S2. Figure S1. Purification of CpMan5B mutant proteins and CbMan5D. CpMan5B, mutant proteins of CpMan5B, and CbMan5D were expressed recombinantly in E. coli and purified from crude cell-free extracts by cobalt metal affinity chromatography. Two μg protein were loaded per well, separated by SDS-PAGE, and stained with Coomassie brilliant blue G-250. Panel A: lane 1, Bio-Rad Broad Range molecular mass standards; lanes 2-9, CpMan5BWT, CpMan5BY12A, CpMan5BY12F, CpMan5BY12Q, CpMan5BR196A, CpMan5BR196H, CpMan5BY12F/R196H, and CpMan5BY12Q/R196H, respectively; lane 10, CbMan5D. Panel B: lane 1 Bio-Rad Broad Range molecular mass standards; lanes 2-8, CpMan5BWT, CpMan5BH84A, CpMan5BH84E, CpMan5BH84M, CpMan5BH84Q, CpMan5BN92A, and CpMan5BN136A, respectively. Figure S2. Immunoblot analyses of CpMan5B mutant proteins and CbMan5D. Purified CpMan5B, mutant proteins of CpMan5B, and CbMan5D were loaded (2 μg protein per lane), separated by SDS-PAGE, transferred to a PVDF membrane and analyzed by Western blot. Panel A: lane 1, Bio-Rad Precision Plus Protein™ Kaleidoscope Standards; lanes 2-9, CpMan5BWT, CpMan5BY12A, CpMan5BY12F, CpMan5BY12Q, CpMan5BR196A, CpMan5BR196H, CpMan5BY12F/R196H, and CpMan5BY12Q/R196H, respectively; lane 10, CbMan5D. Panel B: lane 1, Bio-Rad Precision Plus Protein™ Kaleidoscope Standards; lanes 2-8, CpMan5BWT, CpMan5BH84A, CpMan5BH84E, CpMan5BH84M, CpMan5BH84Q, CpMan5BN92A, and CpMan5BN136A, respectively. (DOCX) [file pone.0080448.s001.docx]

**Expression, purification, and Western blot analyses of purified mannanases**.

Thirteen mutant proteins were purified to near homogeneity (Supplemental Figure S1) with relative molecular weights of ≈41kDa. Purified wild-type and mutant CpMan5B proteins and CbMan5D protein were detected by Western blot analyses [[1](#_ENREF_1)]. Briefly, two micrograms of each purified protein were loaded onto a 12% (w/v) acrylamide gel and subjected to sodium dodecyl sulfate polyacrylamide gel electrophoresis [[2](#_ENREF_2)] and transferred to a polyvinylidene difluoride (PVDF) membrane (GE Healthcare UK Limited, Amersham Place, Little Chalfont, Buckinghamshire, UK). The membrane was blocked with 5% nonfat dry milk in 10mM Tris, 150mM NaCl, 0.05% Tween 20, pH 8.0 (TBST). The primary antibody was a His probe mouse monoclonal IgG_1_ antibody (Santa Cruz Biotechnology, Inc., Santa Cruz, CA) diluted 2000-fold in 1% milk in TBST, and the secondary antibody was alkaline phosphatase-conjugated goat anti-mouse IgG (Sigma Aldrich, St. Louis, MO), diluted 5000-fold in 1% milk in TBST. The hexa-histidine tagged proteins were detected with 3-mL WesternBlue^®^ Stabilized Substrate for Alkaline Phosphatase (Promega Corporation, Madison, WI). A small amount of protein of ≈20kDa was isolated with each variant. Western blot analyses revealed that the ≈20kDa protein contained a histidine tag and was therefore either a proteolytic breakdown product of the recombinant protein or a mis-translated protein fragment (Supplemental Figure S2).

**SUPPLEMENTAL FIGURE LEGENDS**

**Supplemental Figure S1.** Purification of CpMan5B mutant proteins and CbMan5D. CpMan5B, mutant proteins of CpMan5B, and CbMan5D were expressed recombinantly in *E. coli* and purified from crude cell-free extracts by cobalt metal affinity chromatography. Two μg protein were loaded per well, separated by SDS-PAGE, and stained with Coomassie brilliant blue G-250. Panel A: lane 1, Bio-Rad Broad Range molecular mass standards; lanes 2-9, CpMan5B^WT^, CpMan5B^Y12A^, CpMan5B^Y12F^, CpMan5B^Y12Q^, CpMan5B^R196A^, CpMan5B^R196H^, CpMan5B^Y12F/R196H^, and CpMan5B^Y12Q/R196H^, respectively; lane 10, CbMan5D. Panel B: lane 1 Bio-Rad Broad Range molecular mass standards; lanes 2-8, CpMan5B^WT^, CpMan5B^H84A^, CpMan5B^H84E^, CpMan5B^H84M^, CpMan5B^H84Q^, CpMan5B^N92A^, and CpMan5B^N136A^, respectively.

**Supplemental Figure S2.** Immunoblot analyses of CpMan5B mutant proteins and CbMan5D. Purified CpMan5B, mutant proteins of CpMan5B, and CbMan5D were loaded (2 μg protein per lane), separated by SDS-PAGE, transferred to a PVDF membrane and analyzed by Western blot [[1](#_ENREF_1)]. Panel A: lane 1, Bio-Rad Precision Plus Protein™ Kaleidoscope Standards; lanes 2-9, CpMan5B^WT^, CpMan5B^Y12A^, CpMan5B^Y12F^, CpMan5B^Y12Q^, CpMan5B^R196A^, CpMan5B^R196H^, CpMan5B^Y12F/R196H^, and CpMan5B^Y12Q/R196H^, respectively; lane 10, CbMan5D. Panel B: lane 1, Bio-Rad Precision Plus Protein™ Kaleidoscope Standards; lanes 2-8, CpMan5B^WT^, CpMan5B^H84A^, CpMan5B^H84E^, CpMan5B^H84M^, CpMan5B^H84Q^, CpMan5B^N92A^, and CpMan5B^N136A^, respectively.

SUPPLEMENTAL FIGURES

**SUPPLEMENTAL FIGURE S1**

**
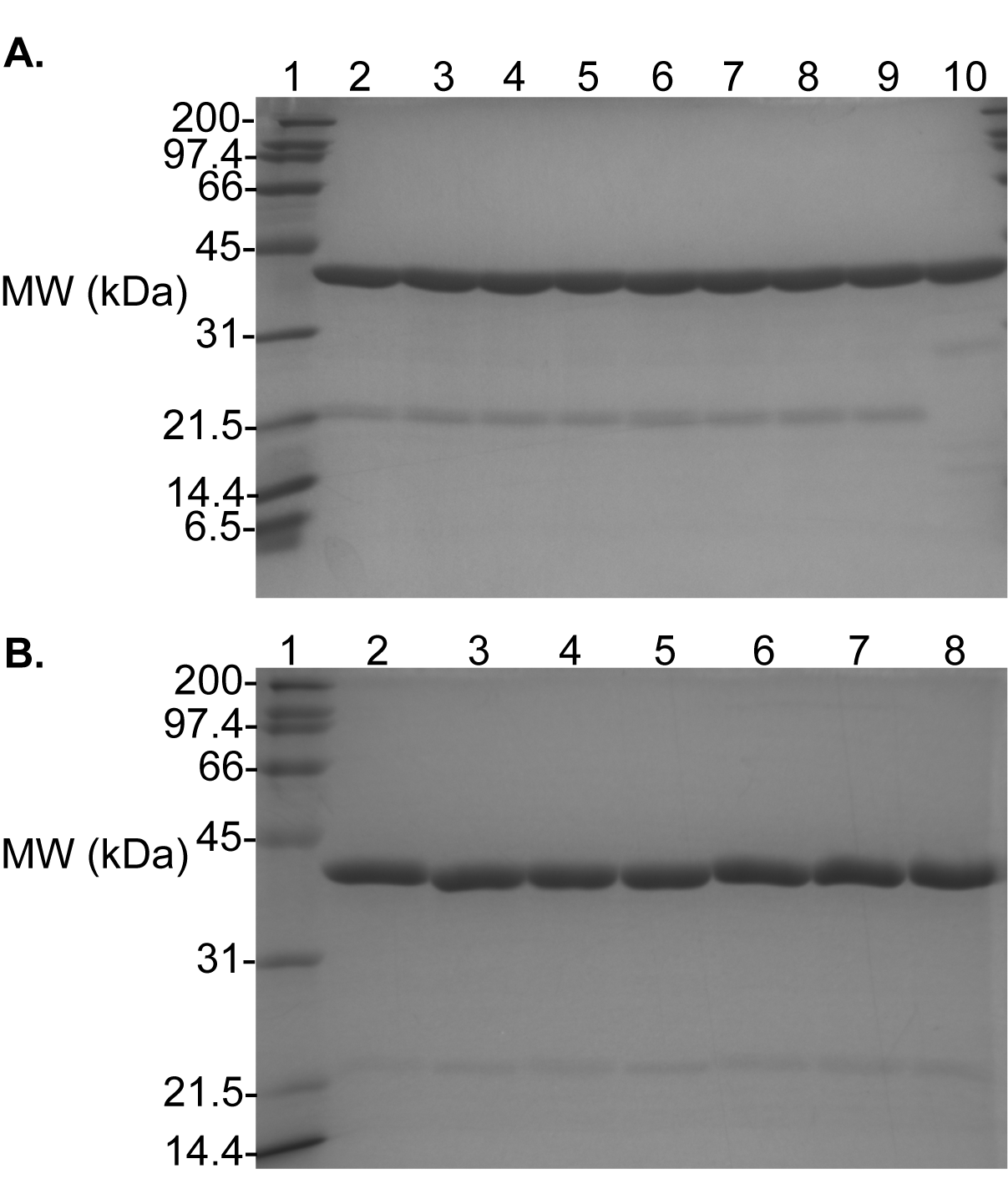
**

**SUPPLEMENTAL FIGURE S2**

**
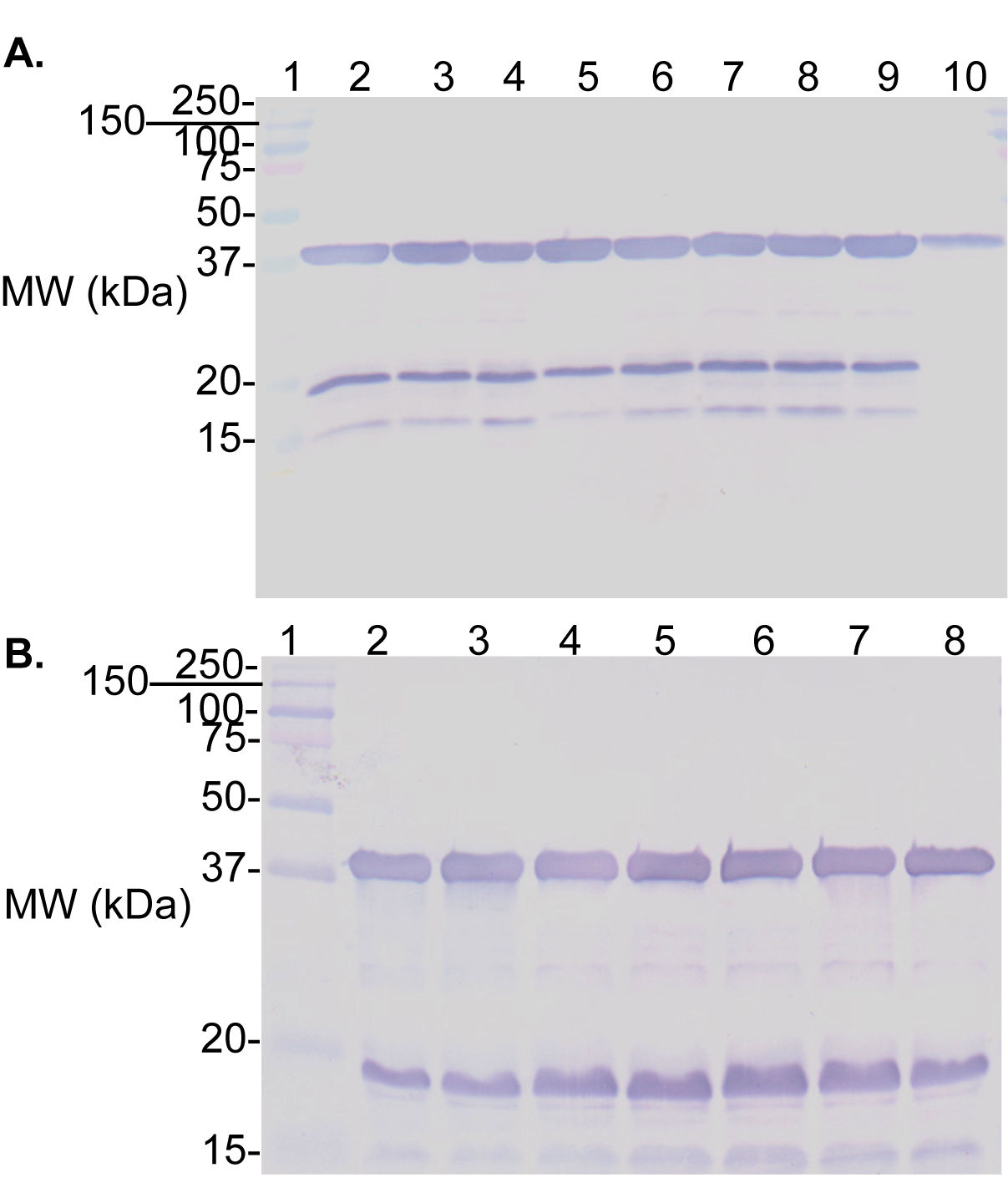
**

**SUPPLEMENTAL REFERENCES**

1. Harlow E, Lane D (1988) Antibodies, a laboratory manual: Cold Spring Harbor Laboratory Press, Cold Spring Harbor, NY.

2. Laemmli UK (1970) Cleavage of structural proteins during the assembly of the head of bacteriophage T4. Nature 227: 680-685.
