# Supplementary material for: Mutational and Structural Analyses of Caldanaerobius polysaccharolyticus Man5B Reveal Novel Active Site Residues for Family 5 Glycoside Hydrolases
Source: PLoS One. 2013 Nov 20;8(11):e80448. doi: 10.1371/journal.pone.0080448 (PMC3835425; doi:10.1371/journal.pone.0080448)
Supplement: File S2 — Figure S3. Figure S3. Activity of CbMan5D with cello- and manno-oligosaccharides as detected by thin layer chromatography. CbMan5D (2.5μM) was reacted with 5 mg mL-1 substrate for 12 hours at 75 °C. One microliter of the reaction products was spotted in each lane. Minus (-) and plus (+) signs indicate the absence or presence of CbMan5D, respectively. Panel A shows the reactions containing manno-configured saccharides (M1-M6). Panel B shows reaction products of gluco-configured saccharides (G1-G6). Standards containing 1 μg each oligosaccharide (M1-M6, Panel A; G1-G6, Panel B) were loaded in lanes at both ends of the plate. (DOCX) [file pone.0080448.s002.docx]

**Activity profile of CbMan5D**.

Enzymatic activities of recombinant CbMan5D were determined at 75 °C in 50 mM sodium citrate buffer containing 150 mM NaCl with pH 5.5 (adjusted at ambient temperature). Manno- and cello-oligosaccharides (G1-G6, M1-M6) were used as substrates. For thin-layer chromatography (TLC) assays, enzyme was diluted to 25 μM in citrate buffer, and 1 μL was added to 9 μL of a 5.56 mg substrate mL^-1^ citrate buffer to obtain final concentrations of 2.5 μM enzyme and 5 mg substrate mL^-1^ in a total volume of 10 μL. The mixtures were reacted for 12 hours and then heat-inactivated for 10 minutes at 100 °C. The hydrolysis products were separated and visualized according to a TLC method that was slightly modified from previously published methods [[1](#_ENREF_1),[2](#_ENREF_2)]. Briefly, 1 μL of each reaction was spotted onto a Silica Gel 60 F254 TLC plastic plate (Merck, Whitehouse Station, NJ), allowed to dry, and resolved by three ascents with a mobile phase of *n*-butanol, acetic acid, and water in a volumetric ratio of 10:5:1. One microliter of a mixture of standards (1mg mL^-1^ M1-M6, G1-G6) was also spotted and resolved in a single lane. The plates were visualized by spraying with a mixture of 50% methanol (v/v), orcinol (0.1% [m/v]), and 10% sulfuric acid (v/v) followed by heating at 80 °C for 10-20 minutes [[3](#_ENREF_3)].

**SUPPLEMENTAL FIGURE S3**

**
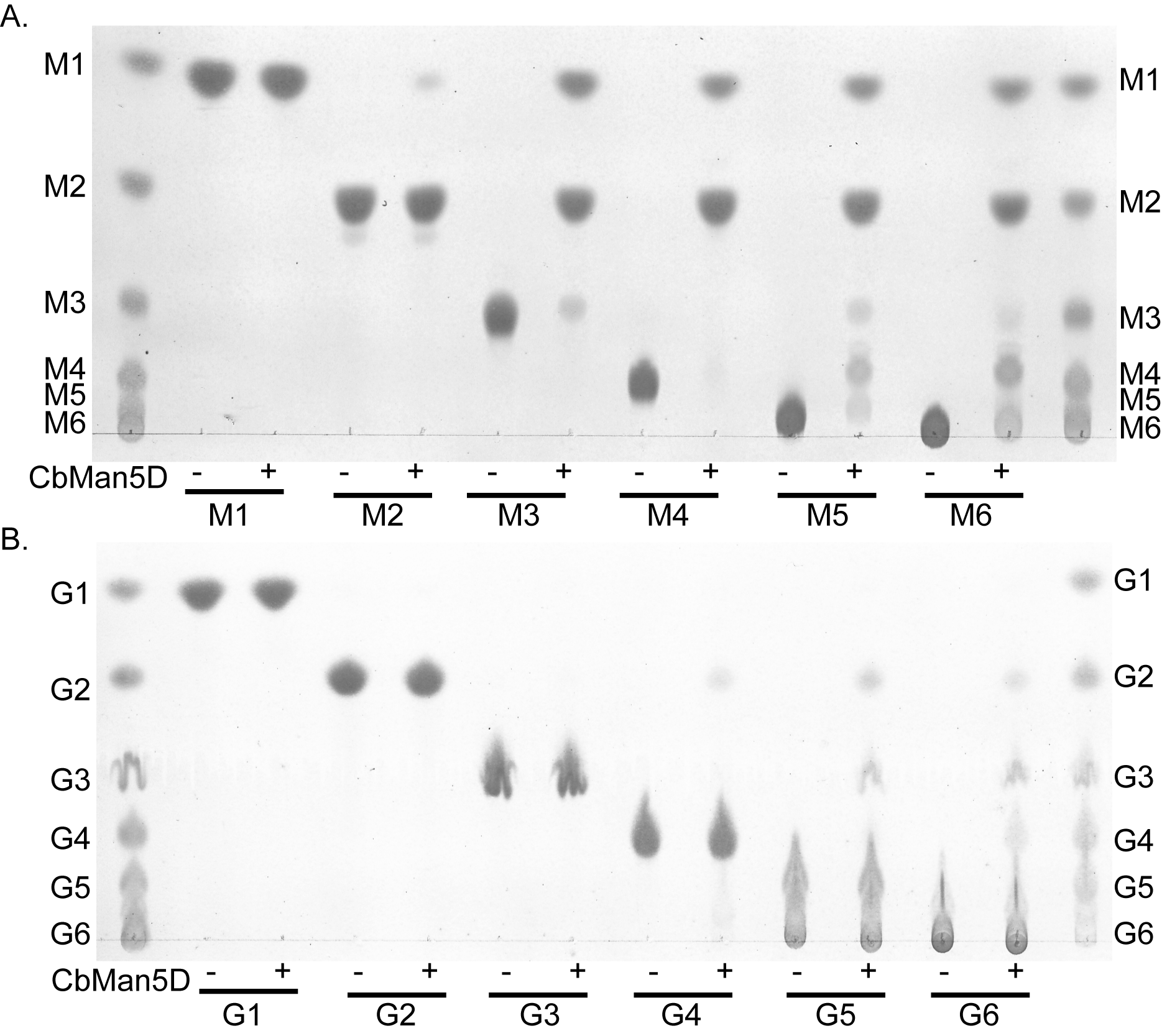
**

**Supplemental Figure S3**. Activity of CbMan5D with cello- and manno-oligosaccharides as detected by thin layer chromatography. CbMan5D (2.5μM) was reacted with 5 mg/mL substrate for 12 hours at 75 °C. One microliter of the reaction products was spotted in each lane. Minus (-) and plus (+) signs indicate the absence or presence of CbMan5D, respectively. Panel A shows the reactions containing manno-configured saccharides (M1-M6). Panel B shows reaction products of gluco-configured saccharides (G1-G6). Standards containing 1 μg each oligosaccharide (M1-M6, Panel A; G1-G6, Panel B) were loaded in lanes at both ends of the TLC plate.

**SUPPLEMENTAL REFERENCES**

1. Kurokawa J, Hemjinda E, Arai T, Kimura T, Sakka K, et al. (2002) *Clostridium thermocellum* cellulase CelT, a family 9 endoglucanase without an Ig-like domain or family 3c carbohydrate-binding module. Appl Microbiol Biotechnol 59: 455-461.

2. Han Y, Dodd D, Hespen CW, Ohene-Adjei S, Schroeder CM, et al. (2010) Comparative analyses of two thermophilic enzymes exhibiting both beta-1,4 mannosidic and beta-1,4 glucosidic cleavage activities from *Caldanaerobius polysaccharolyticus*. J Bacteriol 192: 4111-4121.

3. Dodd D, Kocherginskaya SA, Spies MA, Beery KE, Abbas CA, et al. (2009) Biochemical analysis of a β-D-xylosidase and a bifunctional xylanase-ferulic acid esterase from a xylanolytic gene cluster in *Prevotella ruminicola* 23. J Bacteriol 191: 3328-3338.
