# Supplementary material for: Mutational and Structural Analyses of Caldanaerobius polysaccharolyticus Man5B Reveal Novel Active Site Residues for Family 5 Glycoside Hydrolases
Source: PLoS One. 2013 Nov 20;8(11):e80448. doi: 10.1371/journal.pone.0080448 (PMC3835425; doi:10.1371/journal.pone.0080448)
Supplement: File S3 — Supplementary Tables S1, S2, S3, S4, S5. Table S1. Primer sequence used for gene cloning and mutagenesis. Table S2. Protein properties used for determining protein concentrations. Table S3. Analysis of CD spectra for CpMan5B WT and mutant proteins using DICHROWEB. Table S4. Effects of different mutations on specific activities compared with that of the wild-type CpMan5B protein. Table S5. Comparison of residues in TmCel5A that affect dual mannanase/endoglucanase activity to residues of GH5_36 enzymes. (DOCX) [file pone.0080448.s003.docx]

**SUPPLEMENTAL TABLES**

| **SUPPLEMENTAL TABLE S1.** Primer sequence used for gene cloning and mutagenesis | | |
| --- | --- | --- |
| Primer name | Primers | |
|  | Orientation | Sequence (5'→3') *^a^* |
| Man5B Y12A*^bd^* | Forward | 5'-GCTTTAACTTCCAATGGATGGCCGTATGGGAGGAAGGAAGAG-3' |
| Man5B Y12F*^bd^* | Forward | 5'-GGCTTTAACTTCCAATGGATGTTCGTATGGGAGGA-3' |
| Man5B Y12Q*^bd^* | Forward | 5'- CTTTAACTTCCAATGGATGCAGGTATGGGAGGAAGGAAGAG -3' |
| Man5B H84A*^cd^* | Forward | 5'-GAACATTCACATGTGTTTGAATTTGGCCAGGGCTCCTGGTTAT-3' |
|  | Reverse | 5'-ATAACCAGGAGCCCTGGCCAAATTCAAACACATGTGAATGTTC-3' |
| Man5B H84E*^cd^* | Forward | 5'-GAACATTCACATGTGTTTGAATTTGGAGAGGGCTCCTGGTTATT-3' |
|  | Reverse | 5'-AATAACCAGGAGCCCTCTCCAAATTCAAACACATGTGAATGTTC-3' |
| Man5B H84M*^cd^* | Forward | 5'-CTAGGAACATTCACATGTGTTTGAATTTGATGAGGGCTCCTGGTTATTGC-3' |
|  | Reverse | 5'-GCAATAACCAGGAGCCCTCATCAAATTCAAACACATGTGAATGTTCCTAG-3' |
| Man5B H84Q*^cd^* | Forward | 5'-ATTCACATGTGTTTGAATTTGCAGAGGGCTCCTGGT-3' |
|  | Reverse | 5'-ACCAGGAGCCCTCTGCAAATTCAAACACATGTGAAT-3' |
| Man5B N92A*^cd^* | Forward | 5'-GCACAGGGCTCCTGGTTATTGCATAGCCCGCAACGATATA-3' |
|  | Reverse | 5'-TATATCGTTGCGGGCTATGCAATAACCAGGAGCCCTGTGC-3' |
| Man5B N136A*^cd^* | Forward | 5'-GTTTCTAGCAAGTTTTTAAGCTTTGATCTCGTAGCTGAACCTCCTAATATCGG-3' |
|  | Reverse | 5'-CCGATATTAGGAGGTTCAGCTACGAGATCAAAGCTTAAAAACTTGCTAGAAAC-3' |
| Man5B R196A*^bd^* | Forward | 5'-GTGTTGTGCACAGCGGCGCAGGTTATCAACCCATGG-3' |
| Man5B R196H*^bd^* | Forward | 5'-GGTGTTGTGCACAGCGGCCATGGTTATCAACCCATGGCT-3' |
| Cb234F*^e^* | Forward | 5'-*GACGACGACAAG*ATGAATAAATTACCAAAATACAAAGGATTCAATCTAC-3' |
| Cb234R*^e^* | Reverse | 5'-*GAGGAGAAGCCCGG*TTAAAATCTCATCAAAAGTTCGAGTAAC-3' |
| *^a^* Oligonucleotide primers were synthesized by Integrated DNA Technologies (Coralville, IA). | | |
| *^b^* Primers were designed using the QuikChange^®^ Primer Design Program from Stratagene (La Jolla, CA) with the QuikChange Multi parameters and were synthesized by Integrated DNA Technologies (Coralville, IA). | | |
| *^c^* Primers were designed using the QuikChange^®^ Primer Design Program from Stratagene (La Jolla, CA) with the QuikChange II parameters and were synthesized by Integrated DNA Technologies (Coralville, IA). | | |
| *^d^* Codons selected for introduction of site-specific mutations are underlined. | | |
| *^e^* Nucleotides added for ligation-independent cloning into pET-46b Ek/LIC vector (Novagen, San Diego, CA) are italicized. | | |

| **SUPPLEMENTAL TABLE S2.** Protein properties used for determining protein concentrations*^a^* | | |
| --- | --- | --- |
| Protein name | Molecular mass (kDa) | Extinction coefficient (mM^-1^ cm^-1^) |
| CpMan5B^WT^ | 40.90 | 103.04 |
| CpMan5B^Y12A^ | 40.81 | 101.55 |
| CpMan5B^Y12F^ | 40.88 | 101.55 |
| CpMan5B^Y12Q^ | 40.87 | 101.55 |
| CpMan5B^H84A^ | 40.83 | 103.04 |
| CpMan5B^H84E^ | 40.89 | 103.04 |
| CpMan5B^H84M^ | 40.89 | 103.04 |
| CpMan5B^H84Q^ | 40.89 | 103.04 |
| CpMan5B^N92A^ | 40.86 | 103.04 |
| CpMan5B^N136A^ | 40.86 | 103.04 |
| CpMan5B^R196A^ | 40.82 | 103.04 |
| CpMan5B^R196H^ | 40.88 | 103.04 |
| CpMan5B^Y12F/R196H^ | 40.87 | 101.55 |
| CpMan5B^Y12Q/R196H^ | 40.85 | 101.55 |
| CbMan5D | 40.65 | 90.55 |
| *^a^* Protein parameters were calculated using the Protparam tool on the ExPASy website (http://web.expasy.org/protparam/). | | |

**Circular Dichroism Spectroscopy.**

Circular dichroism scans of CpMan5B^WT^ and the thirteen mutant proteins were carried out as earlier reported [[1](#_ENREF_1)] using a J-815 CD spectropolarimeter (Jasco, Japan) equipped with a constant-temperature cell-holder. Briefly, recombinant proteins in phosphate buffer (10 mM sodium phosphate, pH 5.5) were diluted to 0.2 mg mL^-1^. The protein samples (300 μL) were transferred to a 1-mm quartz cuvette and kept at 25 °C for 5 minutes before scanning. Measurements began with initial wavelength of 260 nm and progressed to 190 nm in wavelength steps of 0.1 nm. The analyses of protein secondary structure were performed using the Dichroweb website using algorithm CDSSTR with reference Set 4 optimized for 190-240 nm [[2](#_ENREF_2),[3](#_ENREF_3)]. Values were calculated from three individual scans. Circular dichroism spectroscopy revealed no obvious changes in secondary structure of any variant in comparison to the wild-type enzyme (Supplemental Table S3).

| **SUPPLEMENTAL TABLE S3.** Analysis of CD spectra for CpMan5B WT and mutant proteins using DICHROWEB*^a^* | | | | | | |
| --- | --- | --- | --- | --- | --- | --- |
| Protein | Helix1 | Helix2 | Strand1 | Strand2 | Turns | Unordered |
| WT | 0.19±0.01 | 0.14±0.01 | 0.12±0.01 | 0.08±0.00 | 0.21±0.01 | 0.28±0.01 |
| Y12A | 0.18±0.02 | 0.14±0.01 | 0.12±0.02 | 0.08±0.01 | 0.20±0.01 | 0.28±0.01 |
| Y12F | 0.17±0.02 | 0.13±0.01 | 0.13±0.01 | 0.09±0.01 | 0.21±0.01 | 0.28±0.00 |
| Y12Q | 0.20±0.02 | 0.16±0.01 | 0.10±0.00 | 0.07±0.01 | 0.20±0.01 | 0.28±0.01 |
| H84A | 0.18±0.01 | 0.13±0.01 | 0.12±0.00 | 0.08±0.00 | 0.20±0.01 | 0.29±0.01 |
| H84E | 0.19±0.01 | 0.14±0.00 | 0.12±0.01 | 0.08±0.01 | 0.20±0.00 | 0.29±0.01 |
| H84M | 0.19±0.02 | 0.14±0.00 | 0.11±0.01 | 0.08±0.01 | 0.20±0.01 | 0.28±0.01 |
| H84Q | 0.19±0.01 | 0.13±0.01 | 0.12±0.01 | 0.08±0.00 | 0.20±0.01 | 0.28±0.01 |
| N92A | 0.19±0.01 | 0.14±0.01 | 0.11±0.02 | 0.08±0.01 | 0.21±0.01 | 0.28±0.01 |
| N136A | 0.19±0.01 | 0.14±0.01 | 0.10±0.01 | 0.07±0.00 | 0.20±0.01 | 0.29±0.01 |
| R196A | 0.17±0.01 | 0.14±0.02 | 0.12±0.02 | 0.08±0.01 | 0.21±0.00 | 0.28±0.01 |
| R196H | 0.17±0.03 | 0.13±0.01 | 0.13±0.01 | 0.08±0.01 | 0.20±0.01 | 0.28±0.01 |
| Y12F/R196H | 0.17±0.02 | 0.14±0.01 | 0.12±0.01 | 0.08±0.01 | 0.20±0.01 | 0.28±0.01 |
| Y12Q/R196H | 0.19±0.02 | 0.15±0.01 | 0.11±0.01 | 0.07±0.01 | 0.20±0.01 | 0.28±0.02 |
| *^a^* CD spectra were recorded in the far-UV range utilizing a J-815 CD spectropolarimeter. The spectra were recorded from 190 to 260 nm at a scanning rate of 50 nm/s and a 1-nm wavelength step with five accumulations. Each spectrum was measured three times, and the data are means ± standard deviations of the means. The spectra were uploaded onto the DICHROWEB online server and analyzed as described previously [[4](#_ENREF_4)]. | | | | | | |
|  |  |  |  |  |  |  |

| **SUPPLEMENTAL TABLE S4.** Effects of different mutations on specific activities compared with that of the wild-type CpMan5B protein*^a^* | | | | | | | | | |
| --- | --- | --- | --- | --- | --- | --- | --- | --- | --- |
| Protein | Rates of product formation from M6 as percentages of wild-type | | | Rates of product formation from G6 as percentages of wild-type | | | Normalized ratios of rates of product formation | | |
|  | M1 | M2 | M3 | G1 | G2 | G3 | M1/G1 | M2/G2 | M3/G3 |
| Wild-type | 100 | 100 | 100 | 100 | 100 | 100 | 1.0 | 1.0 | 1.0 |
| Y12A | 25 | 28 | 32 | 59 | 69 | 70 | 0.42 | 0.41 | 0.45 |
| Y12F | 59 | 63 | 66 | 24 | 30 | 31 | 2.5 | 2.1 | 2.1 |
| Y12Q | 17 | 22 | 23 | 16 | 20 | 21 | 1.0 | 1.1 | 1.1 |
| R196A | n.d. | n.d. | n.d. | n.d. | n.d. | n.d. | n.a. | n.a. | n.a. |
| R196H | 16 | 9 | 16 | 15 | 4 | 4 | 1.0 | 2.4 | 4.1 |
| Y12F/R196H | 9 | n.d. | 7 | 4 | n.d. | n.d. | 2.3 | n.a. | n.a. |
| Y12Q/R196H | n.d. | n.d. | n.d. | n.d. | n.d. | n.d. | n.a. | n.a. | n.a. |
| *^a^* Rates of product formation (nmol min^-1^ mg^-1^ enzyme) are expressed as percentages of the rate from the wild-type enzyme and also as ratios of percent activity for each enzyme variant for the two substrate types (M/G). Abbreviations: n.d. not detected; n.a. not applicable due to non-detectable activity. | | | | | | | | | |
|  |  |  |  |  |  |  |  |  |  |

| SUPPLEMENTAL TABLE S5. Comparison of residues in TmCel5A that affect dual mannanase/endoglucanase activity to residues of GH5_36 enzymes | | | |
| --- | --- | --- | --- |
| Enzyme | **TmCel5A** | **CpMan5B** | **CbMan5D** |
| GH5 Subfamily | **25** | **36** | **36** |
| Residue | N20 | Q9 | L13 |
|  | E23 | Y12 | F16 |
|  | P53 | P42 | P43 |
|  | H95 | H84 | H84 |
|  | H96 | R85 | G85 |
|  | E287 | N292 | N292 |

**SUPPLEMENTAL REFERENCES**

1. Su X, Agarwal V, Dodd D, Bae B, Mackie RI, et al. (2010) Mutational insights into the roles of amino acid residues in ligand binding for two closely related family 16 carbohydrate binding modules. J Biol Chem 285: 34665-34676.

2. Johnson WC (1999) Analyzing protein circular dichroism spectra for accurate secondary structures. Proteins: Structure, Function, and Bioinformatics 35: 307-312.

3. Whitmore L, Wallace BA (2008) Protein secondary structure analyses from circular dichroism spectroscopy: methods and reference databases. Biopolymers 89: 392-400.

4. Dodd D, Kiyonari S, Mackie RI, Cann IK (2010) Functional diversity of four glycoside hydrolase family 3 enzymes from the rumen bacterium *Prevotella bryantii* B14. J Bacteriol 192: 2335-2345.
