# Supplementary material for: Mutational and Structural Analyses of Caldanaerobius polysaccharolyticus Man5B Reveal Novel Active Site Residues for Family 5 Glycoside Hydrolases
Source: PLoS One. 2013 Nov 20;8(11):e80448. doi: 10.1371/journal.pone.0080448 (PMC3835425; doi:10.1371/journal.pone.0080448)
Supplement: File S4 — RCSB PDB Validation Report. (PDF) [file pone.0080448.s004.pdf]

PDB ID : 3W0K
RCSB ID : RCSB095733
TITLE   : Crystal Structure of a glycoside hydrolase
AUTHORS : T.Oyama, H.Nakamura, K.Morikawa, I.K.O.Cann

The following geometrical and stereochemical features have been calculated for your structure.

CLOSE CONTACTS

==> Close contacts in same asymmetric unit. Distances smaller than 2.2 Angstroms are considered as close contacts.

none

==> Close contacts based on crystal symmetry. Distances smaller than 2.2 Angstroms are considered as close contacts.

none

BOND DISTANCES AND ANGLES

Bond and angle checks are performed by first computing the average rms error for all bonds and angles relative to standard values for nucleotide units [L. Clowney et al., Geometric Parameters in Nucleic Acids: Nitrogenous Bases, J.Am.Chem.Soc. 1996, 118, 509−518; A. Gelbin et al., Geometric Parameters in Nucleic Acids: Sugar and Phosphate Constituents, J.Am.Chem.Soc. 1996, 118, 519−529] and amino acid units [R.A. Engh and R. Huber, Structure quality and target parameters, International Tables for Crystallography, Volume F, 2001, 382−392]. Any bond or angle which deviates from the dictionary values by more than six times this computed rms error is identified as an outlier.

==> Covalent Bond Lengths:

The overall RMS deviation for covalent bonds relative to the standard dictionary is   0.007 Angstroms

All covalent bonds lie within a 6.0*RMSD range about the standard dictionary values.

==> Covalent Angle Values:

The overall RMS deviation for covalent angles relative to the standard dictionary is   1.3 degrees.

All covalent bond angles lie within a 6.0*RMSD range about the standard dictionary values.

TORSION ANGLES

The torsion angle distributions have been checked. To view these reports, please refer to the ADIT Validation Server at http://deposit.pdb.org/validate.

==> The following table contains a list of torsion angles outside the expected Ramachandran regions [GJ. Kleywegt and TA. Jones, PHI/PSI−chology: Ramachandran Revisited, Structure 1996, 4, 1395 − 1400].

Residue	Chain	Sequence	PSI	PHI
GLN	A	9	32.79	−98.76
GLU	A	137	76.85	30.33

PHE	A	293	−62.93	−101.69
GLN	B	9	32.51	−98.68
GLU	B	137	77.83	30.33
PHE	B	293	−64.90	−102.50
GLU	B	301	53.72	38.41

CHIRALITY

The chirality has been checked. O1P, O2P, and hydrogen atoms which do not follow the convention defined in the IUBMB (Liebecq, C. Compendium of Biochemical Nomenclature and Related Documents, 2nd ed.; Portland Press: London and Chapel Hill, 1992) and IUPAC nomenclature (J.L. Markley, A. Bax, Y. Arata, C.W. Hilbers, R. Kaptein, B.D. Sykes, P.E. Wright and K. Wuthrich, Recommendations for the Presentation of NMR Structures of Proteins and Nucleic Acids, Pure & Appl. Chem., Vol. 70, pp. 117−142, 1998) have been standardized. Any other stereochemical violations are listed below.

none

SOLVENT

The following solvent molecules are further than 3.5 Angstroms away from macromolecule atoms in the asymmetric unit that are available for hydrogen bonding. Solvent molecules in extended hydration shells separated by 3.5 Angstroms or less are not listed.

```
HETATM 5680  O   HOH A 707      80.011 −59.991  22.798  1.00 28.13      DIST =   3.55 A
HETATM 5697  O   HOH A 724      83.823 −58.003  41.286  1.00 35.15      DIST =   3.62 A
HETATM 5746  O   HOH A 773      49.633 −66.440  11.773  1.00 37.44      DIST =   3.51 A
HETATM 5774  O   HOH A 801      58.639 −57.981  34.003  1.00 32.41      DIST =   4.51 A
```

We have replaced the coordinates for solvent molecules which could be translated back into the asymmetric unit. Please review all solvent molecules in your file and contact us if you have any serious objections.

MISSING RESIDUES

==> The following residues are missing:
(Note: The SEQ number starts from 1 for each chain according to SEQRES sequence record.)

```
RES MOD#C SEQ

ASP(    B 212 )
GLY(    B 213 )
HIS(    B 214 )
LYS(    B 215 )
GLY(    B 216 )
```

PDB Chain_ID: B

```
        1                                                         15
SEQRES: MET LYS LYS TYR GLY PHE ASN PHE GLN TRP MET TYR VAL TRP GLU
COORDS: MET LYS LYS TYR GLY PHE ASN PHE GLN TRP MET TYR VAL TRP GLU
        1                                                         15

        16                                                        30
SEQRES: GLU GLY ARG GLU PRO GLU PRO PRO ASP LYS LYS ALA LEU ASP PHE
COORDS: GLU GLY ARG GLU PRO GLU PRO PRO ASP LYS LYS ALA LEU ASP PHE
        16                                                        30

        31                                                        45
SEQRES: LEU ALA GLU THR GLY PHE ASN PHE VAL ARG ILE PRO VAL ASP TYR
COORDS: LEU ALA GLU THR GLY PHE ASN PHE VAL ARG ILE PRO VAL ASP TYR
        31                                                        45

        46                                                        60
SEQRES: ARG PHE TRP THR ARG ASN PHE ASP TYR PHE ASN PRO ASP LYS LYS
```

```
COORDS: ARG PHE TRP THR ARG ASN PHE ASP TYR PHE ASN PRO ASP LYS LYS
        46                                                       60

        61                                                       75
SEQRES: VAL PHE GLU TYR ILE ASP LEU TYR LEU ARG GLU CYS SER ALA ARG
COORDS: VAL PHE GLU TYR ILE ASP LEU TYR LEU ARG GLU CYS SER ALA ARG
        61                                                       75

        76                                                       90
SEQRES: ASN ILE HIS MET CYS LEU ASN LEU HIS ARG ALA PRO GLY TYR CYS
COORDS: ASN ILE HIS MET CYS LEU ASN LEU HIS ARG ALA PRO GLY TYR CYS
        76                                                       90

        91                                                      105
SEQRES: ILE ASN ARG ASN ASP ILE GLU ARG ASP ASN LEU TRP LEU ASP LYS
COORDS: ILE ASN ARG ASN ASP ILE GLU ARG ASP ASN LEU TRP LEU ASP LYS
        91                                                      105

        106                                                     120
SEQRES: ARG ALA GLN ASP GLY PHE VAL TYR GLN TRP GLU LEU PHE ALA LYS
COORDS: ARG ALA GLN ASP GLY PHE VAL TYR GLN TRP GLU LEU PHE ALA LYS
        106                                                     120

        121                                                     135
SEQRES: ARG TYR LYS GLY VAL SER SER LYS PHE LEU SER PHE ASP LEU VAL
COORDS: ARG TYR LYS GLY VAL SER SER LYS PHE LEU SER PHE ASP LEU VAL
        121                                                     135

        136                                                     150
SEQRES: ASN GLU PRO PRO ASN ILE GLY GLN TYR GLY LEU THR ARG GLU ASN
COORDS: ASN GLU PRO PRO ASN ILE GLY GLN TYR GLY LEU THR ARG GLU ASN
        136                                                     150

        151                                                     165
SEQRES: HIS ALA SER LEU ILE ILE ARG THR VAL GLU ALA ILE ARG LYS ILE
COORDS: HIS ALA SER LEU ILE ILE ARG THR VAL GLU ALA ILE ARG LYS ILE
        151                                                     165

        166                                                     180
SEQRES: ASP PRO ASP ARG GLU ILE VAL ILE ASP GLY LEU GLY GLY GLY ASN
COORDS: ASP PRO ASP ARG GLU ILE VAL ILE ASP GLY LEU GLY GLY GLY ASN
        166                                                     180

        181                                                     195
SEQRES: ILE ALA MET PRO GLU LEU ALA HIS LEU GLY VAL VAL HIS SER GLY
COORDS: ILE ALA MET PRO GLU LEU ALA HIS LEU GLY VAL VAL HIS SER GLY
        181                                                     195

        196                                                     210
SEQRES: ARG GLY TYR GLN PRO MET ALA LEU THR HIS TYR GLN ALA SER TRP
COORDS: ARG GLY TYR GLN PRO MET ALA LEU THR HIS TYR GLN ALA SER TRP
        196                                                     210

        211                                                     225
SEQRES: TRP ASP GLY HIS LYS GLY LEU PRO GLU PRO TYR TYR PRO ASP LEU
COORDS: TRP   ?   ?   ?   ?   ? LEU PRO GLU PRO TYR TYR PRO ASP LEU
        211                                                     225

        226                                                     240
SEQRES: LEU TRP GLN GLY LYS VAL TRP ASN LYS ASP THR LEU ARG GLU TYR
COORDS: LEU TRP GLN GLY LYS VAL TRP ASN LYS ASP THR LEU ARG GLU TYR
        226                                                     240

        241                                                     255
SEQRES: TYR LYS PRO TRP ARG ASP LEU GLN GLN LYS GLY VAL ASN VAL HIS
COORDS: TYR LYS PRO TRP ARG ASP LEU GLN GLN LYS GLY VAL ASN VAL HIS
        241                                                     255

        256                                                     270
SEQRES: ILE GLY GLU PHE GLY CYS PHE ASN LYS THR SER ASN ASP VAL ALA
COORDS: ILE GLY GLU PHE GLY CYS PHE ASN LYS THR SER ASN ASP VAL ALA
        256                                                     270

        271                                                     285
SEQRES: ILE ARG TRP PHE GLU ASP VAL LEU SER LEU TYR LYS GLU PHE GLU
```

```
   COORDS: ILE ARG TRP PHE GLU ASP VAL LEU SER LEU TYR LYS GLU PHE GLU
           271                                                     285

           286                                                     300
   SEQRES: TRP GLY TYR SER LEU TRP ASN PHE LYS GLY PRO PHE GLY ILE VAL
   COORDS: TRP GLY TYR SER LEU TRP ASN PHE LYS GLY PRO PHE GLY ILE VAL
           286                                                     300

           301                                                     315
   SEQRES: GLU HIS GLY ARG PRO GLY ALA LYS TYR GLU TYR TYR ARG GLY PHE
   COORDS: GLU HIS GLY ARG PRO GLY ALA LYS TYR GLU TYR TYR ARG GLY PHE
           301                                                     315

           316                                                     330
   SEQRES: LYS VAL ASP ARG GLU LEU LEU ASP LEU LEU VAL GLU ASN ARG VAL
   COORDS: LYS VAL ASP ARG GLU LEU LEU ASP LEU LEU VAL GLU ASN ARG VAL
           316                                                     330


    MISSING ATOMS
    -------------

==> The following residues have missing atoms:

     RES MOD#C SEQ          ATOMS

     MET(    A   1)          CG   SD   CE
     GLU(    A  15)          CG   CD  OE1  OE2
     HIS(    A 188)          CG  ND1  CD2  CE1  NE2
     ASP(    A 212)          CG  OD1  OD2
     LYS(    A 215)          CG   CD   CE   NZ
     GLN(    A 249)          CG   CD  OE1  NE2
     LYS(    A 250)          CG   CD   CE   NZ
     LYS(    A 308)          CG   CD   CE   NZ
     LYS(    A 316)          CG   CD   CE   NZ
     MET(    B   1)          CG   SD   CE
     GLU(    B  15)          CG   CD  OE1  OE2
     LYS(    B  26)          CG   CD   CE   NZ
     GLU(    B 149)          CG   CD  OE1  OE2
     ASP(    B 235)          CG  OD1  OD2
     LYS(    B 250)          CG   CD   CE   NZ
     LYS(    B 308)          CG   CD   CE   NZ
     TYR(    B 311)          CG  CD1  CD2  CE1  CE2   CZ   OH
     LYS(    B 316)          CG   CD   CE   NZ
```
