# Supplementary material for: Mutational and Structural Analyses of Caldanaerobius polysaccharolyticus Man5B Reveal Novel Active Site Residues for Family 5 Glycoside Hydrolases
Source: PLoS One. 2013 Nov 20;8(11):e80448. doi: 10.1371/journal.pone.0080448 (PMC3835425; doi:10.1371/journal.pone.0080448)
Supplement: File S5 — RCSB PDB Validation Report Summary. (PDF) [file pone.0080448.s005.pdf]

CONFIDENTIAL VALIDATION REPORT

wwPDB Validation Report

PDB ID: 3W0K
RCSB ID: RCSB095733
TITLE: Crystal Structure of a glycoside hydrolase
AUTHOR(S): T.Oyama, H.Nakamura, K.Morikawa, I.K.O.Cann

Summary of PDB Entry Validation

The results of the validation of this PDB entry are shown below. No major issues were raised during data processing.

Geometry Validation

1. **Atomic Clashes**

No issues found.

2. **Peptide Linkage**

No issues found.

3. **Covalent Geometry**

No issues found.

4. **Chirality Error**

No issues found.

CONFIDENTIAL VALIDATION REPORT

Individual Residue Outliers on Real Space R-value

No issues found.

Sequence Validation

The reported biological sequence shows no discrepancy with UniProt sequence (code D9J0D7).

The reported biological sequence and the sequence given in the coordinates show no discrepancy.

Biological Assembly

The biological assembly predicted by PISA is a monomer. This agrees with author's annotation.

Ligand Chemistry

Ligand chemistry has been checked against the Chemical Component Dictionary. The following is a summary.

CONFIDENTIAL VALIDATION REPORT

Identifier: TRS
Name: 2-AMINO-2-HYDROXYMETHYL-PROPANE-1,3-DIOL
Synonym: TRIS BUFFER
Formula: C4 H12 N O3

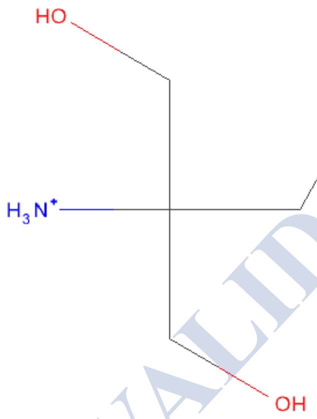

Type	Program	Version	Descriptor
SMILES	ACDLabs	10.04	OCC([NH3+])(CO)CO
SMILES CANONICAL	CACTVS	3.341	[NH3+]C(CO)(CO)CO
SMILES	CACTVS	3.341	[NH3+]C(CO)(CO)CO
SMILES CANONICAL	OpenEye OEToolkits	1.5.0	C(C(CO)(CO)[NH3+])O
SMILES	OpenEye OEToolkits	1.5.0	C(C(CO)(CO)[NH3+])O
InChI	InChI	1.03	InChI=1S/C4H11NO3/c5-4(1-6,2-7)3-8/h6-8H,1-3,5H2/p+1
InChIKey	InChI	1.03	LENZDBCJOHFCAS-UHFFFAOYSA-O

CONFIDENTIAL VALIDATION REPORT

Summary of Structure Factor Validation

Structure quality	
Average Real space R-factor (Calculated by SFCHECK, V7.02.4)	0.0512
Average Real space R-factor (Calculated by MAPMAN, V7.8.5)	0.0917
Average Real-space correlation coefficient (Calculated by SFCHECK, V7.02.4)	0.9927
Average Real-space correlation coefficient (Calculated by MAPMAN, V7.8.5)	0.9394
Average Occupancy-weighted avg temperature factor	16.66

Resolution	
High Resolution (Author reported)	1.60
High Resolution (Calculated by SFCHECK, V7.02.4)	1.60
High Resolution (Calculated by REFMAC, V5.5.0109)	1.600
Low Resolution (Author reported)	19.14
Low Resolution (Calculated by SFCHECK, V7.02.4)	19.14
Low Resolution (Calculated by REFMAC, V5.5.0109)	19.136

Crystal data	
Space group	P 1 21 1
Total number of reflections	100324
Number of reflections used	95254

R-factors	
R-factor (Author reported)	0.176
R-factor (Calculated by SFCHECK, V7.02.4)	0.180
R-factor (Calculated by REFMAC, V5.5.0109)	0.1757
Free R-factor (Author reported)	0.188
Free R-factor (Calculated by SFCHECK, V7.02.4)	0.190
Free R-factor (Calculated by REFMAC, V5.5.0109)	0.1862

Wilson statistics (PHENIX, V1.6-289)	

Wilson B-factor	13.94
Wilson Scale	-1.97

**Padilla-Yeates statistics for twin detection (PHENIX, V1.6-289)**	
Padilla-Yeates <|L|>	0.491
Padilla-Yeates <L*L>	0.322

CONFIDENTIAL VALIDATION REPORT
